# Association of antiretroviral therapy with high-risk human papillomavirus, cervical intraepithelial neoplasia, and invasive cervical cancer in women living with HIV: a systematic review and meta-analysis

**DOI:** 10.1016/S2352-3018(17)30149-2

**Published:** 2017-10-26

**Authors:** Helen Kelly, Helen A Weiss, Yolanda Benavente, Silvia de Sanjose, Philippe Mayaud, ART and HPV Review Group, You-lin Qiao, Rui-Mei Feng, Hugo DeVuyst, Vanessa Tenet, Antoine Jaquet, Deborah Konopnicki, Tanvier Omar, Lynette Menezes, Corinna Moucheraud, Risa Hoffman

**Affiliations:** a Clinical Research Department, London School of Hygiene & Tropical Medicine, London, UK; b MRC Tropical Epidemiology Group, London School of Hygiene & Tropical Medicine, London, UK; c Cancer Epidemiology Research Program, Catalan Institute of Oncology, IDIBELL, L'Hospitalet de Llobregat, Barcelona, Spain; d CIBER en Epidemiología y Salud Pública (CIBERESP), Barcelona, Spain

## Abstract

**Background:**

The interactions between antiretroviral therapy (ART) and high-risk human papillomavirus (HPV) and cervical lesions in women living with HIV are poorly understood. We reviewed the association of ART with these outcomes.

**Methods:**

We did a systematic review and meta-analysis by searching MEDLINE and Embase databases for cross-sectional or cohort studies published in English between Jan 1, 1996, and May 6, 2017, which reported the association of ART with prevalence of high-risk HPV or prevalence, incidence, progression, or regression of histological or cytological cervical abnormalities, or incidence of invasive cervcal cancer. Studies were eligible if they reported the association of combination ART or highly active ART use with the following outcomes: high-risk HPV prevalence; squamous intraepithelial lesion (SIL) or cervical intraepithelial neoplasia (CIN) prevalence, incidence, progression, or regression; and invasive cervical cancer incidence among women living with HIV. We did random-effects meta-analyses to estimate summary statistics. We examined heterogeneity with the *I*^2^ statistic. This review is registered on the PROSPERO database at the Centre of Reviews and Dissemination, University of York, York, UK (registration number CRD42016039546).

**Findings:**

We identified 31 studies of the association of ART with prevalence of high-risk HPV (6537 women living with HIV) and high grade cervical lesions (HSIL-CIN2+; 9288 women living with HIV). Women living with HIV on ART had lower prevalence of high-risk HPV than did those not on ART (adjusted odds ratio [aOR] 0·83, 95% CI 0·70–0·99; *I*^2^=51%, adjusted for CD4 cell count and ART duration), and there was some evidence of association with HSIL-CIN2+ (0·65, 0·40–1·06; *I*^2^=30%). 17 studies reported the association of ART with longitudinal cervical lesion outcomes. ART was associated with a decreased risk of HSIL-CIN2+ incidence among 1830 women living with HIV (0·59, 0·40–0·87; *I*^2^=0%), SIL progression among 6212 women living with HIV (adjusted hazard ratio [aHR] 0·64, 95% CI 0·54–0·75; *I*^2^=18%), and increased likelihood of SIL or CIN regression among 5261 women living with HIV (1·54, 1·30–1·82; *I*^2^=0%). In three studies among 15 846 women living with HIV, ART was associated with a reduction in invasive cervical cancer incidence (crude HR 0·40, 95% CI 0·18–0·87, *I*^2^=33%).

**Interpretation:**

Early ART initiation and sustained adherence is likely to reduce incidence and progression of SIL and CIN and ultimately incidence of invasive cervical cancer. Future cohort studies should aim to confirm this possible effect.

**Funding:**

UK Medical Research Council.

## Introduction

Cervical cancer is the most common cancer affecting women in low-income and middle-income countries,^1^ and one of the most common cancers in women living with HIV.^2^ Women living with HIV have higher prevalence of genital high-risk oncogenic human papillomavirus (HPV) infection than do the general population,^3^ they are also more likely to have persistent infection^4^ and progression of cervical intraepithelial neoplasia (CIN) lesions.^5^ As combined antiretroviral therapy (ART) is scaled up, the effect on cervical cancer due to longer survival is unknown.

The interactions of ART and the natural history of high-risk HPV and cervical lesions in women living with HIV are poorly understood. Observational studies differ with respect to study design, outcomes, timing of ART initiation and effectiveness of ART use, making it difficult to estimate the true effect of ART. Previous systematic reviews have explored the association of ART and high-risk HPV and cervical lesions,^5^, ^6^, ^7^ but to our knowledge no meta-analysis has quantified the risk of high-risk HPV infection and cervical lesions among ART users compared with ART-naive women. In view of the large and increasing number of women on ART, improved understanding of the interplay of ART, immune recovery, and virological control on the natural history of high-risk HPV infection and CIN progression is needed to guide screening programmes.

**Research in context**

**Evidence before this study**

Women living with HIV have higher prevalence of genital high-risk oncogenic human papillomavirus (HPV) infection than the general population and are more likely to have persistent infection and progression of cervical intraepithelial neoplasia (CIN) lesions. Increased access to antiretroviral therapy (ART) has increased the life expectancy of women living with HIV, but many remain susceptible to high-risk HPV incidence and persistence and cervical lesion incidence and progression. The precise effect of ART on the natural history of high-risk HPV infection and cervical lesion progression is not well established, and studies evaluating this association have reported conflicting results. We searched all available publications in English in the MEDLINE and Embase databases from Jan 1, 1996, to May 6, 2017, which reported the association of ART with prevalence of high-risk HPV or prevalence, incidence, progression, or regression of histological (CIN) or cytological (squamous intraepithelial lesions [SIL]) cervical abnormalities, or incidence of invasive cervical cancer. We found 31 studies of the association of ART with prevalence of high-risk HPV (6537 women living with HIV), and CIN of grade 2 or higher (CIN2+) diagnosed by histology or high-grade SIL (HSIL+) diagnosed by cytology only (9288 women living with HIV). Furthermore, 17 studies reported the association of ART with longitudinal cervical lesion outcomes (any CIN or SIL), providing data for 6864 women living with HIV, and three studies reported the association of ART with incidence of invasive cervical cancer among 15 826 women living with HIV.

**Added value of this study**

We found that prevalence of high-risk HPV and histology diagnosed HSIL-CIN2+ was lower among ART users compared with those not on treatment. ART was associated with a decreased risk of histology diagnosed HSIL-CIN2+ incidence, cytology diagnosed SIL incidence, and SIL progression. Women living with HIV on ART had an increased likelihood of histology diagnosed CIN or cytology diagnosed SIL regression and a decreased risk of invasive cervical cancer incidence. To our knowledge, this is the first study to quantify the effect of ART on prevalent high-risk HPV, high-grade cervical lesion outcomes, and invasive cervical cancer in a meta-analysis. Studies that adjusted for either nadir or current CD4 cell count and time-varying effects of ART were more likely to show a protective effect of ART on these outcomes. Studies from Africa and Europe or North America provide indication that ART was associated with lower prevalence of high-risk HPV and cervical lesions, and over prolonged duration, ART can prevent cervical lesion incidence and progression, promote regression, and prevent incidence of invasive cervical cancer. Fewer studies exist from Asia and Latin America with the majority being cross-sectional in design, and these studies were less likely to report any protective association of ART. Because some studies from Latin America have reported an increased risk of high-risk HPV and CIN2+ among women with a lower nadir CD4 cell count, the lack of association might reflect the timing of ART in relation to HPV infection and cervical lesion development in these populations. Our findings highlight the importance of early ART initiation (before reaching a low nadir CD4 cell count) and sustained effectiveness, as evidenced by duration, high adherence, virological control, and CD4 cell recovery, in controlling HPV infection and cervical disease progression.

**Implications of all the available evidence**

The current recommendation of encouraging earlier ART initiation, coupled with rapid virological control, and sustained adherence is likely to lead to an earlier and possibly more functionally complete mucosal immune reconstitution. ART users with low or unknown nadir CD4 cell count should be screened frequently because their risk of high-risk HPV infection and cervical lesion progression remains high. Longitudinal studies in the era of immediate unconditional ART initiation should capture the greater benefit of ART treatment on cervical disease and cancer.

We aimed to review and to summarise the literature about the association of ART with high-risk HPV prevalence, and with cervical lesion prevalence, incidence, progression and regression, and invasive cervical cancer incidence. We also aimed to investigate the role of HIV-related cofactors that might modify these associations, such as ART duration, timing of treatment initiation, immune suppression, and recovery.

## Methods

### Search strategy and selection criteria

We searched MEDLINE and Embase databases for publications in English with search terms for human papillomavirus, CIN, SIL, invasive cervical cancer, and ART (appendix p 1). Reference lists of review articles and all articles identified in the systematic search were checked. We did the search from Jan 1, 1996 (when highly active ART came into use), up to May 6, 2017. One author (HK) screened all abstracts. Two authors (HK and PM) obtained full-text copies of relevant publications, assessed them for eligibility, and reached consensus on potential relevance.

Studies were eligible if they reported the association of combination ART or highly active ART use (referred to as ART from now on) with the following outcomes: prevalence of high-risk HPV; prevalence, incidence, progression, or regression of SIL diagnosed with cytology or CIN diagnosed with histology; and incidence of invasive cervical cancer among women living with HIV. We also considered studies eligible if they provided raw data to calculate an unadjusted effect estimate.

For high-risk HPV outcomes, we included studies reporting genital high-risk HPV. There were no exclusions on HPV test methods. For the prevalent lesion outcomes, studies reporting cervical lesions using visual inspection with acetic acid or Lugol's iodine but without high-resolution colposcopy were excluded because of the poor sensitivity and specificity of visual inspection alone in detecting high-grade lesions.

For prevalent outcomes, cross-sectional studies were included if they reported the association of ART use with high-risk HPV or any grade of histological or cytological cervical lesion. Cohort studies were included if participants initiated ART at enrolment, were followed up, and had measures of high-risk HPV at baseline and in the follow-up visit.

For the longitudinal outcomes, we included cohort studies reporting the association of ART with the incidence, progression, and regression of any CIN grade diagnosed by histology or any SIL grade diagnosed by cytology (which could include atypical squamous cells of undetermined significance as well as low-grade and high-grade lesions) because SIL represent various incremental degrees of high-risk HPV persistence and subsequent lesion development. Only cohort studies examining invasive cervical cancer incidence among ART users and treatment-naive women in the ART era were included because they provide the most robust direct comparison of the effect of therapy on invasive cervical cancer.

For publications that reported results from the same cohort, but at different follow-up visits, the publication that gave the most relevant description of the cohort and study design and the most complete set of results was included. There was no restriction on age or geographical location.

### Data extraction

From the consensus list, one author (HK) extracted the data and a second author (HAW) checked a random sample of 25%. For studies reporting prevalence of high-risk HPV or cervical lesions, odds ratios (ORs) were extracted. For studies reporting cervical lesion incidence, progression or regression, hazard ratios (HRs) or ORs were extracted.

### Methodological quality assessment

We assessed studies primarily on adjustment for HIV-related factors (current and nadir CD4 cell count and ART duration). We considered cross-sectional studies that adjusted for either current or nadir CD4 cell count or ART duration separately in sensitivity analyses, as were cohort studies that adjusted for time on ART during follow-up. We also assessed study quality by participant selection, statistical method, HPV test used, and cervical lesion (cytological or histological) classification (appendix pp 6–18).

### Statistical analysis

We did meta-analyses for the discrete outcomes of high-risk HPV prevalence, high-grade lesion (high-grade squamous intraepithelial lesion or cervical intraepithelial neoplasia grade 2 or higher, diagnosed by cytology or histology [HSIL-CIN2+]) prevalence, incidence, progression and regression of any histology diagnosed CIN or cytology diagnosed SIL, and incidence of invasive cervical cancer.

We report adjusted effect estimates when available. For the cross-sectional studies in which adjusted effect estimates were not reported but raw data were provided, we calculated crude ORs (HK) and independently verified them (HAW and PM). We contacted authors when the paper suggested that relevant data were collected but not reported.

We used random-effects meta-analysis to estimate pooled effects to account for between-study heterogeneity.^8^ We examined heterogeneity using the *I*^2^ statistic and publication bias using funnel plots and Begg's test for correlation between the effect estimate and their variances.^9^, ^10^ We did an influence analysis to assess the robustness of the pooled summary effects by excluding each of the studies from the pooled estimate. We did subgroup analyses by geographical region to compare pooled effects and heterogeneity. We did sensitivity analyses excluding studies unadjusted for HIV-related factors. We analysed data using Stata version 14.

This review is reported according to the Preferred Reporting Items for Systematic Reviews and Meta-Analyses (PRISMA)^11^ and the Meta-analysis Of Observational Studies in Epidemiology (MOOSE) guidelines.^12^ The review protocol and the dataset are available online.

### Role of the funding source

There was no funding source for this study. The corresponding author had full access to all the data in the study and had final responsibility for the decision to submit for publication.

## Results

**Figure 1:**
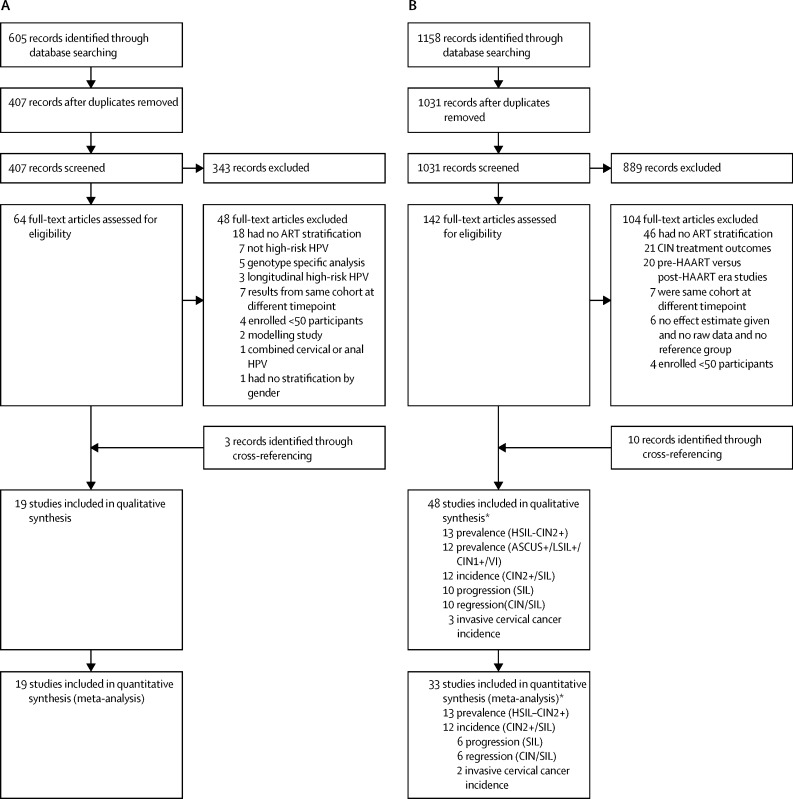
Study selection for outcomes of high-risk HPV (A) and cervical lesions (B) HPV=human papillomavirus. HAART=highly active antiretroviral therapy. CIN=cervical intraepithelial neoplasia. SIL=squamous intraepithelial lesions. HSIL=high-grade SIL. LSIL=low-grade SIL. ASCUS= atypical squamous cells of undetermined significance. *Some studies contributed to more than one outcome (ie, incidence and progression, or progression and regression). Individual studies are summarised in table 1.

We identified 605 publications for the association of ART and high-risk HPV prevalence through MEDLINE and Embase searches, 198 of which were duplicates and removed; and we excluded 343 after abstract review, leaving 64 articles for full-text review. Finally, 16 articles matched inclusion criteria and we identified three additional publications through cross-referencing (figure 1). Data were extracted from 19 publications (12 cross-sectional; seven cohort) representing 20 discrete populations and providing data from 6537 women living with HIV, of whom 3677 (56%) were taking ART (range 19–85% in cross-sectional studies), 2032 (31%) were ART-naive, and 828 (13%) were ART initiators. Four studies^13^, ^14^, ^15^, ^16^ compared high-risk HPV before and after ART initiation (ie, women acted as their own controls; table 1; appendix p 2). One publication provided data from two countries,^17^ and was considered as two individual studies in the analysis, resulting in 20 included studies.

**Table 1:** Summary of studies reporting the association of ART use with high-risk HPV, cervical lesion outcomes and invasive cervical cancer incidence

	**Location**	**Study period**	**Total sample**	**Mean or median age (IQR), years**	**ART users (%)**	**Cervical lesions**	
						Definition	Diagnostic method
**High risk HPV prevalence**							
Zeier et al (2015)^13^	Western Cape, South Africa	2009–11	300	36 (ART); 31 (ART-naive)	68% initiated during follow-up*	··	··
Rositch et al (2013)^14^	Rakai, Uganda	2007–10	96	35 (31–44)	0%*	··	··
Minkoff et al (2010)^15^	5 cities, USA	1994–2002	286	NR	0%*	··	··
Fife et al (2009)^16^	Puerto Rico/USA	2001–05	146	35	0%*	··	··
Kelly et al (2017)^17^	Ouagadougou, Burkina Faso	2011–12	570	36 (31–41)	67%	··	··
Kelly et al (2017)^17^	Johannesburg, South Africa	2011–12	613	34 (30–40)	65%	··	··
Ezechi et al (2014)^18^	Ogun and Lagos, Nigeria	NR	220	37 (31–45)	72%	··	··
Reddy et al (2014)^19^	Lilongwe, Malawi	2011–12	294	36 (30–43)	85%	··	··
De Vuyst et al (2012)^20^	Nairobi, Kenya	2009	497	38	75%	··	··
Jaquet et al (2012)^21^	Abidjan, Côte d'Ivoire	Jun to Oct, 2010	254	36 (32–42)	75%	··	··
Veldhuijzen et al (2011)^22^	Kigali, Rwanda	2006–09	124	27 (23–32)	40%	··	··
Menezes et al (2016)^23^	Chennai, India	July to Aug, 2011	50	33	48%	··	··
Zhang et al (2014)^24^	Yunnan, China	NR	301	34	64%	··	··
Mane et al (2012)^25^	Pune, India	NR	277	33	56%	··	··
Aggarwal et al (2012)^26^	Chandigarh, India	NR	130	34	75%	··	··
Rocha-Brischiliari et al (2014)^27^	Maringa city, Brazil	Apr to Oct, 2011	178	Range: 18–66 years	79%	··	··
Dames et al (2014)^28^	Nassau, Bahamas	Feb to Sep, 2008	165	40	81%	··	··
Grinsztejn et al (2009)^29^	Rio de Janeiro, Brazil	1996–2006	634	36 (29–43)	68%	··	··
Konopnicki et al (2013)^30^	Brussels, Belgium	2002–11	652	38 (31–45)	79%	··	··
Blitz et al (2013)^31^	11 cities, Canada	1993–2002	750	33 (28–38)	19%	··	··
**HSIL-CIN2+ prevalence**							
Kelly et al (2017)^17^	Ouagadougou, Burkina Faso	2011–12	530	36 (31–41)	73%	HSIL-CIN2+	Histology
Kelly et al (2017)^17^	Johannesburg, South Africa	2011–12	566	34 (30–40)	65%	HSIL-CIN2+	Histology
De Vuyst et al (2012)^20^	Nairobi, Kenya	2009	470	38	75%	HSIL-CIN2+	Histology
Memiah et al (2015)^32^	Kiambu, Kenya	2009–10	686	52% <40 years	16%	HSIL-CIN2+	Histology
Huchko et al (2014)^33^	Kisumu, Kenya	2007–10	3185	33 (29–39)	50%	HSIL-CIN2+	Histology
Mabeya et al (2012)^34^	Eldoret, Kenya	NR	149	34	67%	HSIL-CIN2+	Histology
Ezechi et al (2014)^35^	Ogun and Lagos, Nigeria	NR	490	37 (31–45)	76%	HSIL-CIN2+	Cytology
Firnhaber et al (2010)^36^	Johannesburg, South Africa	NR	1010	34 (18–65)	65%	HSIL-CIN2+	Cytology
Mogtomo et al (2009)^37^	Douala, Cameroon	NR	70	35	50%	HSIL-CIN2+	Cytology
Feng et al (2017)†	Yunnan, China	2009	301	34	64%	HSIL-CIN2+	Histology
Sahasrabuddhe et al (2010)^38^	Pune, India	2006–07	271	30 (27–34)	26%	HSIL-CIN2+	Histology
De Andrade et al (2011)^39^	Rio de Janeiro, Brazil	1996–2007	340	34 (28–41)	26%	HSIL-CIN2+	Histology
Patrelli et al (2013)^40^	Parma, Italy	1993–2010	194	41	66%	HSIL-CIN2+	Cytology
Kitchener et al (2007)^41^	6 cities, Europe	2000–04	1026	33	56–79%	HSIL-CIN2+	Cytology
**SIL-CIN incidence**							
Minkoff et al (2010)^15^	5 cities, USA	1994–2002	286	NR	All ART initiators	Normal to ASCUS+	Cytology
Kelly et al (2017)^17^	Johannesburg, South Africa	2011–12	379	34 (30–40)	71% at end of follow-up	<CIN2 to CIN2/3	Histology
Adler et al (2012)^54^	Soweto, South Africa	2003–10	767	33	2% at baseline; 17% initiation during follow-up	Normal to ASCUS	Cytology
Firnhaber et al (2012)^55^	Johannesburg, South Africa	NR	326	35 (31–41)	71% at baseline	Normal to ASCUS+	Cytology
Kreitchmann et al (2013)^56^	Porto Alegre, Brazil	1997–2007	349	32	38%	<LSIL to LSIL+,	Cytology
Sirera et al (2008)^57^	Barcelona, Spain	1997–2006	127	35	71% at baseline	Normal to LSIL+	Cytology
Soncini et al (2007)^58^	Parma, Italy	1993–2003	101	NR	43% through follow-up	Normal to LSIL+	Cytology
Lehtovirta et al (2006)^59^	Helsinki, Finland	1989–2003	55	30–36	48% at baseline; 64% at follow-up	Normal to LSIL+	Cytology
Heard et al (2006)^60^	Paris, France	1993–2005	298	33 (29–38)	49% through follow-up	Normal to ASCUS+	Cytology
Schuman et al (2003)^61^	4 cities, USA	1993–95	629	35	33% at baseline	Normal to LSIL+	Cytology
Ellerbrock et al (2000)^62^	New York, USA	1991–96	328	47% <35 years	54% on ≥1 ARV during study period	Normal to ASCUS+	Cytology
Clifford et al (2016)^63^	5 cities, Switzerland	1995–2013	1451	NR	54%	<CIN2 to CIN2/3	Histology
**SIL progression**							
Blitz et al (2013)^31^	11 cities, Canada	1993–2002	326	33 (28–38)	19% at baseline; 64% by study end	ASCUS to any grade higher	Cytology
Adler et al (2012)^54^	Soweto, South Africa	2003–10	1123	33	2% at baseline; 17% initiation during follow-up	Subsequent smear with worsening dysplasia	Cytology
Firnhaber et al (2012)^55^	Johannesburg, South Africa	NR	326	35 (31–41)	71% at baseline	Normal to LSIL+; LSIL to HSIL+	Cytology
Schuman et al (2003)^61^	4 cities, USA	1993–95	629	35	33% at baseline	Normal/ASCUS to LSIL+; LSIL to HSIL	Cytology
Zeier et al (2012)^64^	Western Cape, South Africa	2004–09	1048	33	18%	LSIL to HSIL+	Cytology
Omar et al (2011)^65^	Soweto, South Africa	2003–10	1074	32 (28–37)	6% at baseline; 20% initiated during follow-up	Normal to LSIL+; LSIL to HSIL+/ASC-H	Cytology
Kim et al (2013)^66^	New York, USA	1991–2011	245	37	NR	Normal to ASCUS+; ASCUS to LSIL+	Cytology
Paramsothy et al (2009)^67^	4 cities, USA	1996–2000	537	34	47% during follow-up	Normal to ASCUS; ASCUS to LSIL; LSIL to HSIL	Cytology
Minkoff et al (2001)^68^	6 cities, USA	1994–95	741	37	1% at baseline	Subsequent smear any grade higher than baseline	Cytology
Lillo et al (2001)^69^	Milan, Italy	1995–97	163	34	46% through follow-up	Normal to LSIL+; LSIL to HSIL	Cytology
**SIL or CIN regression**							
Minkoff et al (2010)^15^	5 cities, USA	1994–2002	286	NR	All ART initiators	SIL to lower grade	Cytology
Blitz et al (2013)^31^	11 cities, Canada	1993–2002	326	33 (28–38)	19% at baseline; 64% by study end	≥ASCUS to <ASCUS	Cytology
Adler et al (2012)^54^	Soweto, South Africa	2003–10	1123	33	2% at baseline; 17% initiation during follow-up	Subsequent improvement in cytological results	Cytology
Schuman et al (2003)^61^	4 cities, USA	1993–95	629	35	33% at baseline	LSIL or HSIL to <LSIL	Cytology
Zeier et al (2012)^64^	Western Cape, South Africa	2004–09	1048	33	18%	≥LSIL to <LSIL	Cytology
Paramsothy et al (2009)^67^	4 cities, USA	1996–2000	537	34	47% during follow-up	HSIL to LSIL; LSIL to ASCUS; ASCUS to normal	Cytology
Minkoff et al (2001)^68^	6 cities, USA	1994–95	741	37	1% at baseline	Lower grade abnormality than baseline	Cytology
Massad et al (2004)^70^	6 cities, USA	1994–2002	202	38	22%	CIN1 to normal	Histology
Heard et al (2002)^71^	Paris, France	1993–99	168	33	56% through follow-up	Reversion to normal or from high to low grade	Cytology
Del Mistro et al (2004)^72^	Vicenza and Padova, Italy	1994–2002	201	33	37%	Normal or lower SIL grade at subsequent exam	Cytology
**Invasive cervical cancer incidence**							
Clifford et al (2016)^63^	5 cities, Switzerland	1995–2013	80	NR	54%	<CIN2 to ICC	Unclear
Chen et al (2014)^73^	Taiwan	2000–08	1360	32	28%	Incidence of CIS or ICC	Unclear
Guiguet et al (2009)^74^	62 French university hospitals, France	1998–2006	14 406	39 (35–44)	17%	Incidence of ICC	ICD10

SIL diagnosed by cytology or CIN diagnosed by histology. Detailed description of studies in appendix (pp 2–5). HPV=human papillomavirus. HSIL=high-grade squamous intraepithelial lesion. CIN=cervical intraepithelial neoplasia. ASCUS=atypical squamous cells of undetermined significance. LSIL=low-grade squamous intraepithelial lesion. ARV=antiretroviral. ART=antiretroviral therapy. ASC-H=atypical squamous cells-cannot exclude HSIL. CIS=carcinoma in situ. NR=not reported. ICD10=International Classification of Diseases version 10. ICC=invasive cervical cancer.

* Studies that included women who initiated ART at enrolment.

† Personal communication.

**Figure 2:**
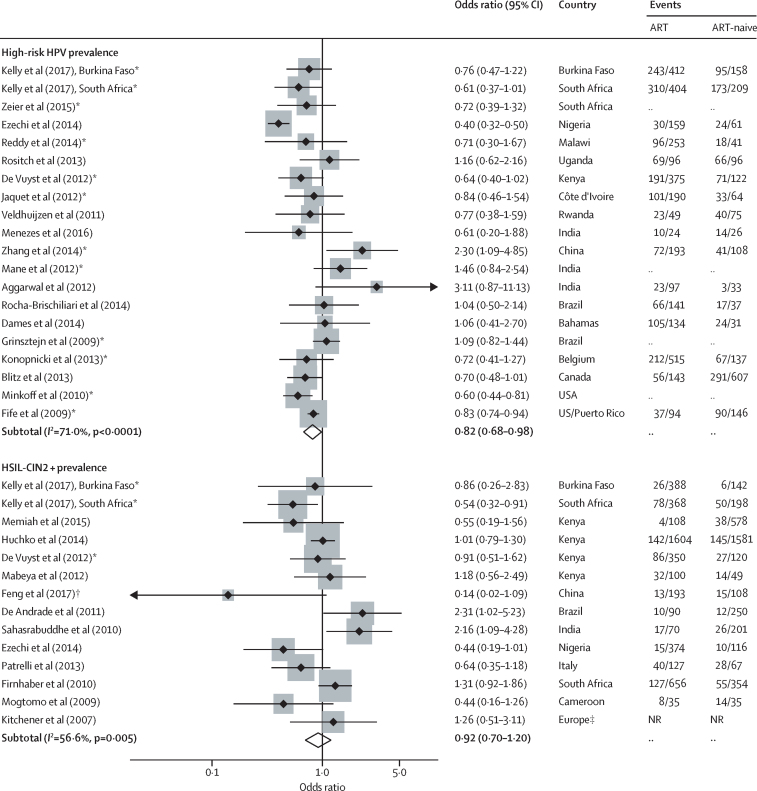
Meta-analysis of the prevalence of high-risk HPV and HSIL-CIN2+ among ART users compared with ART-naive Weights are from random-effects analysis. HPV=human papillomavirus. HSIL=high-grade squamous intraepithelial lesion. CIN2+=cervical intraepithelial lesion, grade 2 or higher. ART=antiretroviral therapy. NR=not reported. *Studies that adjusted for any of ART duration, current or nadir CD4 cell count. †Personal communication. ‡Includes France, Ireland, Italy, Poland, and the UK ((authors report rate ratio of cytology-diagnosed HSIL+ among ART users over follow-up as opposed to odds ratio).

The pooled OR among 20 studies^13^, ^14^, ^15^, ^16^, ^17^, ^18^, ^19^, ^20^, ^21^, ^22^, ^23^, ^24^, ^25^, ^26^, ^27^, ^28^, ^29^, ^30^, ^31^ indicates that women living with HIV on ART had a lower risk of high-risk HPV prevalence compared with women who were ART-naive (crude OR 0·82, 95% CI 0·68–0·98); but there was a high degree of heterogeneity between studies (*I*^2^=71%, p value for heterogeneity<0·0001; table 2, figure 2). Restricting the analysis to the 12 studies that adjusted for either current or nadir CD4 cell count, or ART duration,^13^, ^15^, ^16^, ^17^, ^19^, ^20^, ^21^, ^24^, ^25^, ^29^, ^30^ the OR was similar but with a moderate degree of heterogeneity (adjusted [a] OR 0·85, 95% CI 0·73–1·00, adjusted for nadir or current CD4 cell count; aOR 0·83, 95% CI 0·70–0·99, *I*^2^=51%, p value for heterogeneity=0·02, with additional adjustment for duration on ART). The reduction in heterogeneity on adjustment for confounding was most noticeable among the studies from Africa; among six studies^13^, ^17^, ^19^, ^20^, ^21^ the aOR was 0·70 (95% CI 0·56–0·88) with no evidence of heterogeneity (*I*^2^=0·0%, p=0·97). Similarly, among studies from Europe or North America, three studies^15^, ^16^, ^30^] showed a similar reduction in high-risk HPV (aOR 0·74, 95% CI 0·59–0·93; *I*^2^=48%, p=0·14). This was by contrast with the two studies from Asia^24^, ^25^ (1·72, 1·10–2·68; *I*^2^=0%, p=0·34) and three from Latin America^27^, ^28^, ^29^ (crude OR 1·08, 95% CI 0·84–1·39; *I*^2^=0%, p =0·99).

**Table 2:** Meta-analysis of the association of ART with the prevalence of high-risk HPV and HSIL-CIN2+ among women living with HIV

	Crude analysis*				**Adjusted analysis**†			
	n studies	OR (95%CI)	*I*^2^	p value for heterogeneity	n studies	OR (95%CI)	*I*^2^	p value for heterogeneity
**High-risk HPV prevalence**								
All	20	0·82 (0·68–0·98)	71·0%	<0·0001	12	0·83 (0·70–0·99)	51·0%	0·02
Africa	9	0·67 (0·52–0·88)	58·8%	0·01	6	0·70 (0·56–0·88)	0%	0·97
Asia	4	1·60 (0·93–2·75)	38·6%	0·18	2	1·72 (1·10–2·68)	0%	0·34
Latin America	3	1·08 (0·84–1·39)	0%	0·99	··	··	··	··
Europe or North America	4	0·75 (0·63–0·88)	29·9%	0·23	3	0·74 (0·59–0·93)	48·4%	0·14
**HSIL-CIN2+ prevalence**								
All	14	0·92 (0·70–1·20)	56·6%	0·01	4	0·65 (0·40–1·06)	29·5%	0·25
Africa	9	0·84 (0·64–1·10)	45·5%	0·07	3	0·70 (0·48–1·01)	0%	0·40
Asia	2	0·66 (0·05–9·37)	83·7%	0·01	··	··	··	··
Latin America	1	2·31 (1·02–5·23)	··	··	··	··	··	··
Europe or North America	2	0·83 (0·43–1·57)	32·2%	0·23	··	··	··	··

HPV=human papillomavirus. OR=odds ratio. HSIL-CIN2+=high-grade squamous intraepithelial lesions or cervical intraepithelial neoplasia, grade 2 or higher. ART=antiretroviral therapy.

* Includes studies with no adjustment and studies that adjust for sociodemographic factors only but no adjustment for HIV-related factors.

† Adjusted for at least one of the following: current CD4 cell count, nadir CD4 cell count, and ART duration.

The pooled estimate from four cohort studies that followed women before and after ART initiation^13^, ^14^, ^15^, ^16^ provides strong evidence of a reduced prevalence of high-risk HPV after ART compared with before ART initiation (crude OR 0·80, 95% CI 0·72–0·89; aOR 0·79, 95% CI 0·71–0·88; *I*^2^=48%, p=0·15; data not shown).

Nine studies reported the association of ART duration with high-risk HPV prevalence.^17^, ^19^, ^20^, ^21^, ^23^, ^24^, ^28^, ^30^ Although high-risk HPV prevalence was similar among the ART-naive and short-duration users (<2 years), the pooled OR suggests that prevalence of high-risk HPV was lower among prolonged ART users (≥2 years) than in short-duration users and ART-naive combined (crude OR 0·65, 95% CI 0·55–0·77; *I*^2^=0%, p=0·92; appendix p 19). Among the seven studies adjusted for current and nadir CD4 cell count,^17^, ^19^, ^20^, ^21^, ^24^, ^30^ the association was similar (aOR 0·65, 95% CI 0·55–0·78; *I*^2^=0%, p=0·91, data not shown).

There was no evidence to suggest publication bias (ie, smaller studies were not more likely to report a positive association; Beggs rank correlation test p=0·12 for the crude analysis, p=0·34 for adjusted analysis).

We identified 1158 publications for the association of ART and any cervical lesion outcome, of which 127 duplicates were removed and 889 excluded after abstract review, leaving 142 articles for full review. Finally, we identified 38 articles that matched the inclusion criteria and ten additional publications through cross-referencing (figure 1). Data from an ongoing but unpublished study on association of ART with the prevalence of histology diagnosed HSIL-CIN2+ (Feng et al, 2017) was also included (data provided by Y-L Qiao, personal communication, appendix p 3).

13 studies^17^, ^20^, ^32^, ^33^, ^34^, ^35^, ^36^, ^37^, ^38^, ^39^, ^40^, ^41^ reported the association of ART with the prevalence of cytology or histology diagnosed HSIL-CIN2+ among 9288 women living with HIV, of whom 5161 (56%) were taking ART (range across studies 16% to 79%) and 4127 (44%) were ART-naive (table 1). One publication provided data from two countries,^17^ and was considered as two individual studies in the analysis. 12 further studies reported the association of ART with the prevalence of combined cytology diagnosed outcomes of atypical squamous cells of undetermined significance (or higher),^42^, ^43^, ^44^, ^45^, ^46^, ^47^, ^48^ and low-grade SIL (or higher),^49^ histology diagnosed CIN (grade 1 or higher),^50^, ^51^ and abnormalities on visual inspection with colposcopy (appendix p 20).^52^, ^53^

**Figure 3:**
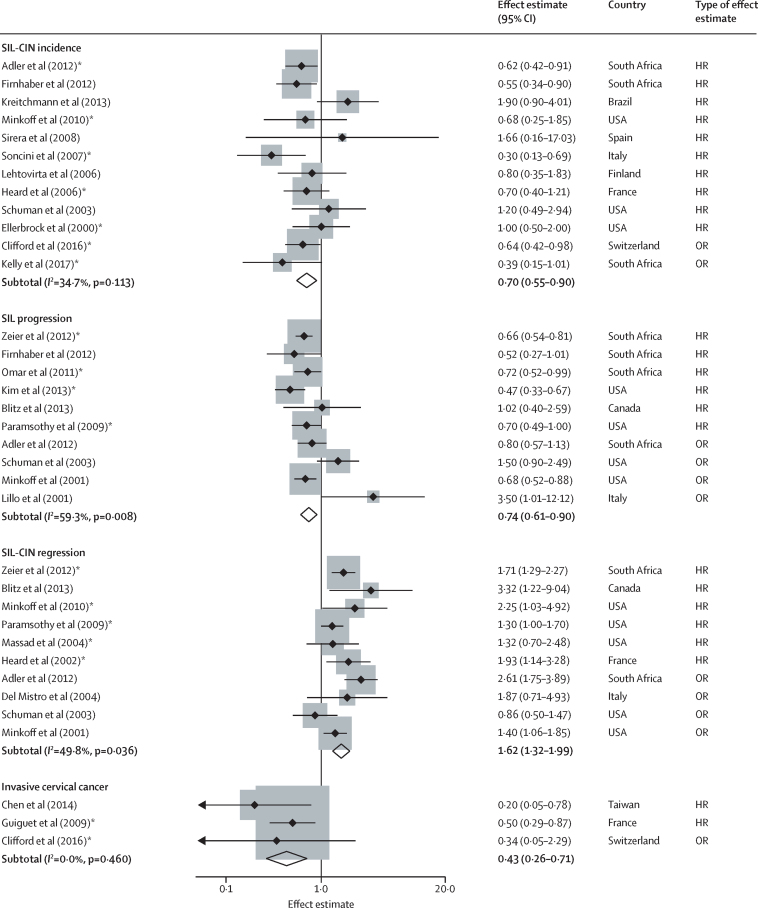
Meta-analysis of cervical lesion incidence, progression and regression, and invasive cervical cancer incidence among ART users compared with ART-naive Weights are from random effects analysis. Only studies that reported HR from time-to-event analysis included in the meta-analysis (table 3). HR=hazard ratio. OR=odds ratio. SIL=squamous intraepithelial lesion. CIN=cervical intraepithelial neoplasia.*Adjusted for the time-varying effects of ART or CD4 cell count.

Ten studies reported the association of ART with cytology diagnosed SIL incidence,^15^, ^54^, ^55^, ^56^, ^57^, ^58^, ^59^, ^60^, ^61^, ^62^ and two studies with histology diagnosed HSIL-CIN2+ incidence^17^, ^63^ from a combined total of 5096 women (table 1). We included ten studies^31^, ^54^, ^55^, ^61^, ^64^, ^65^, ^66^, ^67^, ^68^, ^69^ for cytology diagnosed SIL progression from a combined total of 6212 women, and ten studies^15^, ^31^, ^54^, ^61^, ^64^, ^67^, ^68^, ^70^, ^71^, ^72^ for regression of histology diagnosed CIN or cytology diagnosed SIL from a combined total of 5261 women (table 1). Only one study reported the regression from histological CIN grade 1 to normal.^70^ Three studies^63^, ^73^, ^74^ reported the association of ART with invasive cervical cancer incidence among 15 846 women. Studies reporting the association of ART with cervical lesion incidence, progression and regression, and invasive cervical cancer incidence are summarised in figure 3.

The pooled OR among 14 studies^17^, ^20^, ^31^, ^32^, ^33^, ^34^, ^35^, ^36^, ^37^, ^38^, ^39^, ^40^, ^41^ reporting the association of ART and cervical lesion prevalence suggests no evidence of an association of ART with the prevalence of HSIL-CIN2+ diagnosed by either cytology or histology (crude OR 0·92, 95% CI 0·70–1·20; *I*^2^=56·6%, p=0·01; table 2, figure 2). Restricting the analysis to those studies^17^, ^20^, ^32^, ^33^, ^34^, ^38^, ^39^ with histological confirmation found no evidence of an association (crude OR 0·99, 95% CI 0·69–1·41; *I*^2^=58·7%, p=0·01; data not shown) but when analyses were restricted to studies^17^, ^20^ that adjusted for both current CD4 cell count and ART duration, there was some evidence that ART users had decreased prevalence of HSIL-CIN2+ compared with ART-naive women (aOR 0·85, 95% CI 0·62–1·18; *I*^2^=0%, p=0·56, adjusted for current CD4 cell count alone; aOR 0·65, 95% CI 0·40–1·06; *I*^2^=29·5%, p=0·25, with additional adjustment for duration on ART). Three studies,^17^, ^20^ all from the African region, reported the association of ART duration with prevalent HSIL-CIN2+ diagnosed by histology. The pooled OR suggests that CIN2+ was lower among prolonged ART users (≥2 years) than in short-duration users (<2 years) and ART-naive combined (aOR 0·68, 0·49–0·94; *I*^2^=2.5%, p=0·36, adjusted for age and current CD4 cell count; appendix p 19).

Study size varied widely (range 70–3185 women living with HIV). The largest study^33^ enrolled 3185 women (34% of participants included in the meta-analysis). However, excluding this study did not change the overall results. We found no evidence to suggest publication bias among studies reporting cervical lesion prevalence (Begg's rank correlation test; crude analysis p=0·48, adjusted analysis p=0·50).

An additional sensitivity analysis including low-grade lesion outcomes (atypical squamous cells of undetermined significance or low-grade SIL diagnosed by cytology, CIN [grade 1 or higher] diagnosed by histology, and abnormality on visual inspection with colposcopy) suggests that ART is associated with a reduction in these outcomes, although these associations were not significant (appendix pp 20–22).

The pooled HR among ten studies^15^, ^54^, ^55^, ^56^, ^57^, ^58^, ^59^, ^60^, ^61^, ^62^ reporting the association of ART and cervical lesion incidence provides weak evidence of an association of ART with cytology diagnosed SIL incidence (crude HR 0·75, 95% CI 0·56–1·00; *I*^2^=41%, p =0·09; table 3). Among five studies that adjusted for the time-varying effects of ART,^15^, ^54^, ^58^, ^60^, ^62^ we found evidence of a reduction in SIL incidence among ART users (aHR 0·64, 95% CI 0·47–0·86; *I*^2^=19·4%, p=0·29). There was no evidence to suggest publication bias for these studies (Beggs rank correlation test; crude analysis p=0·42, adjusted analysis p=1·000).

**Table 3:** Meta-analysis of the association of ART with cervical lesion incidence, progression and regression, and invasive cervical cancer incidence among women living with HIV

	Crude analysis*				**Adjusted analysis**†			
	n studies	HR (95%CI)‡	*I*^2^	p value for heterogeneity	n studies	HR (95%CI)‡	*I*^2^	p value for heterogeneity
**SIL incidence**								
All	10	0·75 (0·56–1·00)	40·9%	0·09	5	0·64 (0·47–0·86)	19·4%	0·29
Africa	2	0·59 (0·44–0·80)	0%	0·71	1	0·62 (0·42–0·91)	··	··
Latin America	1	1·90 (0·90–4·01)	··	··	··	··	··	··
Europe or North America	7	0·73 (0·52–1·03)	14·0%	0·32	4	0·64 (0·40–1·02)	39·0%	0·18
**SIL progression**								
All	6	0·64 (0·56–0·74)	0%	0·42	4	0·64 (0·54–0·75)	17·8%	0·30
Africa	3	0·67 (0·56–0·79)	0%	0·68	2	0·68 (0·57–0·80)	0%	0·65
Europe or North America	3	0·62 (0·43–0·90)	46·4%	0·16	2	0·57 (0·39–0·85)	58·0%	0·12
**SIL-CIN regression**								
All	6	1·61 (1·31–1·97)	18·3%	0·30	5	1·54 (1·30–1·82)	0%	0·42
Africa	··	··	··	··	1	1·71 (1·29–2·27)	··	··
Europe or North America	5	1·62 (1·21–2·16)	28·4%	0·23	4	1·45 (1·17–1·81)	1·8%	0·38
**Invasive cervical cancer incidence**								
All	2	0·40 (0·18–0·87)	32·7%	0·22	1	0·50 (0·29–0·87)	··	··

* Includes studies with no adjustment potential confounders and studies that adjust for sociodemographic factors only but no adjustment for HIV related factors.

† Includes studies that adjusted for time-varying ART or time-varying CD4 cell count.

‡ Only studies that reported HR from time-to-event analysis included in the meta-analysis. HR=hazard ratio. SIL=squamous intraepithelial lesions. CIN=cervical intraepithelial neoplasia.

When analyses were restricted to two studies^17^, ^63^ that reported incidence of HSIL-CIN2+ determined by histology, there was strong evidence that prolonged duration ART users had reduced incidence compared with ART-naive women (aOR 0·59, 95% CI 0·40–0·87 [adjusted for nadir CD4 cell count]; *I*^2^=0%, p=0·35, data not shown).

The pooled HR among six studies^31^, ^55^, ^64^, ^65^, ^66^, ^67^ suggests a reduced hazard of cytology diagnosed SIL progression among ART users (crude HR 0·64, 95% CI 0·56–0·74; *I*^2^=0%, p=0·42; table 3). Restricting the analysis to four studies^64^, ^65^, ^66^, ^67^ that adjusted for time-varying ART did not alter the estimate (aHR 0·64, 95% CI 0·54–0·75; *I*^2^=17·8%, p=0·30). Similarly, there was no variation in HR by region.

The pooled HR among six studies^15^, ^31^, ^64^, ^67^, ^70^, ^71^ suggests an increased likelihood of regression of cytology diagnosed SIL or histology diagnosed CIN among ART users (crude HR 1·61, 95% CI 1·31–1·97; *I*^2^=18·3%, p=0·30; table 3). Restricting the analysis to five studies^15^, ^64^, ^67^, ^70^, ^71^ that adjusted for time-varying ART during follow-up did not alter the estimate (aHR 1·54, 95% CI 1·30–1·82; *I*^2^=0%, p=0·42).

Although most studies reported progression or regression of any cytology diagnosed SIL grade, one study reported progression of low-grade SIL to a higher grade, and regression from high-grade to low-grade SIL, diagnosed by cytology.^64^ No change in the estimate was observed when excluding that study for either the progression or regression outcomes. No evidence suggests publication bias for the progression studies (Beggs rank correlation test, p=0·85), but there is some evidence for bias in the regression studies (p=0·04) because more of the small studies report a positive association of ART with regression. However, the largest study (enrolling 1048 women living with HIV followed up over a median 18 months^64^) finds a significant increased likelihood of regression among ART users compared with ART-naive participants (aHR 1·71, 95% CI 1·29–2·27, adjusted for ART duration, age, and excision treatment), suggesting a real beneficial effect of ART.

The pooled HR among two studies^73^, ^74^ suggests a decreased risk of invasive cervical cancer incidence among ART users (crude HR 0·40, 95% CI 0·18–0·87; *I*^2^=32·7%, p=0·22; table 3). There is no evidence to suggest publication bias for these studies (Beggs rank correlation test; p=0·32, data not shown).

## Discussion

Our results indicate that women on ART had a lower prevalence of high-risk HPV and a reduction in the incidence of histology diagnosed HSIL-CIN2+ and invasive cervical cancer, after adjustment for CD4 cell count and treatment duration.

To our knowledge, this is the first meta-analysis to investigate the associations between ART and high-risk HPV and cytology and histology diagnosed cervical lesion and invasive cervical cancer outcomes. Doing a meta-analysis of observational studies for high-risk HPV and cervical lesion outcomes has difficulties because of inherent differences in study populations, definitions of exposure and timescale of outcomes used, and the varying approaches to adjustment of effect estimates. The particular challenge with cross-sectional studies concerns the timing of HPV infection and development of cervical lesions, which might take several years, in relation to ART initiation and immune restoration that can happen more rapidly but is dependent on nadir CD4 cell count. The discordances in natural histories of HPV, CIN, and HIV disease might explain the observed lack of effect of ART on prevalent high-grade cervical lesions in this analysis.

Restricting analyses to those studies that adjusted for nadir or current CD4 cell count or ART duration suggests that ART is associated with a reduction in high-risk HPV or cervical lesion outcomes, with less between-study heterogeneity. In studies that report limited or no association, immune reconstitution by ART might not have been established early enough after HPV infection to prevent or to reverse the development of high-risk HPV persistence or CIN2+. However, prospective studies that adjusted for the time-varying effects of ART use and CD4 cell count suggested a reduction in the incidence of CIN2+ and incidence and progression of SIL.

Several studies reported that a high nadir CD4 cell count was associated with a 36–70% reduced risk of high-risk HPV^29^, ^30^ and a 36–80% reduced risk of CIN2+^33^, ^39^, ^63^ compared with those with low nadir CD4 cell count. Other studies^17^, ^30^ have shown that, once on ART, effective therapy (ie, patients with prolonged duration, sustained HIV-1 viral suppression and stable high CD4 cell count) was associated with a reduction in high-risk HPV persistence and histology diagnosed CIN2+. Further evidence suggests that high-risk HPV prevalence and incidence decreased and cytology diagnosed SIL regression increased in women who were highly adherent to ART.^15^ Of crucial importance, ART is associated with a reduction in incidence of invasive cervical cancer, especially if started at higher nadir CD4 cell count,^63^ and used over longer durations by adherent patients.^73^ This encouraging finding contrasts with previous studies that had shown a paradoxical increase in invasive cervical cancer incidence after the introduction of highly active ART.^75^ This could be because, in the early ART era, therapy was initiated at a lower nadir CD4 cell count, at which full restoration of cervical mucosal immunity was not obtained while life expectancy of patients and their likelihood to develop cancers were higher.

The representation of studies from African settings has been steadily increasing; many of the earlier studies were done in the USA or Europe, leading to a geographical and period heterogeneity. The African studies^17^, ^54^, ^55^, ^64^, ^65^ provide encouraging indication that earlier initiation and effective ART over a prolonged duration can prevent cervical lesion incidence and progression and promote regression. Conversely, we found fewer studies from Latin America and Asia and most were cross-sectional in design. These studies^24^, ^25^, ^26^, ^27^, ^28^, ^29^, ^38^, ^39^ reported an opposite increased risk of high-risk HPV and high-grade cervical lesions among ART users. The lack of prospective studies in these regions prohibits a more direct assessment of the role of ART on longitudinal outcomes. An increased frequency of cervical cancer screening visits remains important especially among women on ART if they have started at a low nadir CD4 cell count. This concerns a generation of women who might have started ART with older guidelines at specific lower CD4 cell count thresholds and who might never have fully recovered their HPV-specific mucosal immune response.

We encountered several limitations in this review. Firstly, most cross-sectional studies used a binary category of ART users and treatment-naive. A more informative analysis would be to measure the effect of ART duration because there is a non-comparability among women initiating ART with decreasing CD4 cell count compared with those with higher CD4 cell count not yet needing treatment. Women who initiate ART are more likely to have advanced HIV disease, lower nadir CD4 cell counts, and higher HIV-1 viral loads than are those who have not yet started ART. The definition of ART-naive participants also varied across studies, which in some cases included women on monotherapy or dual-therapy regimens, and we cannot rule out the possibility that these women could have had lower or less stable CD4 cell counts to justify ART initiation.

The outcome definitions for cervical lesions varied between studies, in particular the use of cytological and histological measurement and definition of progression and regression between grades. Most prospective studies used cytological outcomes instead of the more desirable histological endpoint and grouping of cytology diagnosed grades of SIL varied; this, coupled with the variation in ART exposure between populations (eg, varying regimens and duration), makes interpretation of pooled data less clear. The possibility of unmeasured confounding also exists. Additionally, many studies did not report on likely predictors or effect modifiers of progression or regression of cervical lesions, which include nadir CD4 cell count, ART adherence, and HIV virological control. When available, we did sensitivity analysis that adjusted for time-varying effects of ART. Finally, individual patient-level data meta-analysis would allow for better harmonisation of these definitions and adjustments, which would provide a more precise and robust estimate of the association of ART and high-risk HPV and cervical lesion outcomes.

Our review has practical implications for the management of HIV patients and cervical cancer control worldwide. The current recommendation of encouraging earlier ART initiation, coupled with rapid virological control, and sustained adherence is likely to lead to an earlier and possibly more functionally complete mucosal immune reconstitution. We expect that this should in turn lead to a more rapid clearance of high-risk HPV, thus reducing cytology diagnosed SIL and histology diagnosed CIN incidence or progression and ultimately reducing cervical cancer incidence in this high-risk population. ART users with low or unknown nadir CD4 cell count remain at significant high risk despite ART initiation and should be screened frequently.

## References

[1] Bosch FX, Broker TR, Forman D (2013). Comprehensive control of human papillomavirus infections and related diseases. Vaccine.

[2] Control CfDa (1992). 1993 revised classification system for HIV infection and expanded surveillance case definition for AIDS among adolescents and adults. MMWR Recomm Rep.

[3] McDonald AC, Tergas AI, Kuhn L, Denny L, Wright TC (2014). Distribution of human papillomavirus genotypes among HIV-Positive and HIV-negative women in Cape Town, South Africa. Front Oncol.

[4] Ahdieh L, Klein RS, Burk R (2001). Prevalence, incidence, and type-specific persistence of human papillomavirus in human immunodeficiency virus (HIV)-positive and HIV-negative women. J Infect Dis.

[5] Denslow SA, Rositch AF, Firnhaber C, Ting J, Smith JS (2014). Incidence and progression of cervical lesions in women with HIV: a systematic global review. Int J STD AIDS.

[6] Bratcher LF, Sahasrabuddhe VV (2010). The impact of antiretroviral therapy on HPV and cervical intraepithelial neoplasia: current evidence and directions for future research. Infect Agent Cancer.

[7] Kelly H, Mayaud P, de Sanjose S (2015). Concomitant infection of HIV and HPV: what are the consequences?. Curr Obstet Gynecol Rep.

[8] Harris RJ BM, Deeks JJ, Harbord RM, Altman DG, Sterne JAC (2009). Meta-analysis in Stata: metan, metacum, and metap.

[9] Egger M, Smith GD, Phillips AN (1997). Meta-analysis: principles and procedures. BMJ.

[10] Begg CB, Mazumdar M (1994). Operating characteristics of a rank correlation test for publication bias. Biometrics.

[11] Moher D, Liberati A, Tetzlaff J, Altman DG (2009). Preferred reporting items for systematic reviews and meta-analyses: the PRISMA statement. BMJ.

[12] Stroup DF, Berlin JA, Morton SC (2000). Meta-analysis of observational studies in epidemiology: a proposal for reporting. Meta-analysis Of Observational Studies in Epidemiology (MOOSE) group. JAMA.

[13] Zeier MD, Botha MH, Engelbrecht S (2015). Combination antiretroviral therapy reduces the detection risk of cervical human papilloma virus infection in women living with HIV. AIDS.

[14] Rositch AF, Gravitt PE, Tobian AA (2013). Frequent detection of HPV before and after initiation of antiretroviral therapy among HIV/HSV-2 co-infected women in Uganda. PLoS One.

[15] Minkoff H, Zhong Y, Burk RD (2010). Influence of adherent and effective antiretroviral therapy use on human papillomavirus infection and squamous intraepithelial lesions in human immunodeficiency virus-positive women. J Infect Dis.

[16] Fife KH, Wu JW, Squires KE, Watts DH, Andersen JW, Brown DR (2009). Prevalence and persistence of cervical human papillomavirus infection in HIV-positive women initiating highly active antiretroviral therapy. J Acquir Immune Defic Syndr.

[17] Kelly HA, Sawadogo B, Chikandiwa A (2017). Epidemiology of high-risk human papillomavirus and cervical lesions in African women living with HIV/AIDS: effect of anti-retroviral therapy. AIDS.

[18] Ezechi OC, Ostergren PO, Nwaokorie FO, Ujah IA, Pettersson KO (2014). The burden, distribution and risk factors for cervical oncogenic human papilloma virus infection in HIV positive Nigerian women. Virol J.

[19] Reddy D, Njala J, Stocker P (2015). High-risk human papillomavirus in HIV-infected women undergoing cervical cancer screening in Lilongwe, Malawi: a pilot study. Int J STD AIDS.

[20] De Vuyst H, Mugo NR, Chung MH (2012). Prevalence and determinants of human papillomavirus infection and cervical lesions in HIV-positive women in Kenya. Br J Cancer.

[21] Jaquet A, Horo A, Charbonneau V (2012). Cervical human papillomavirus and HIV infection in women of child-bearing age in Abidjan, Cote d'Ivoire, 2010. Br J Cancer.

[22] Veldhuijzen NJ, Braunstein SL, Vyankandondera J (2011). The epidemiology of human papillomavirus infection in HIV-positive and HIV-negative high-risk women in Kigali, Rwanda. BMC Infect Dis.

[23] Menezes LJ, Poongulali S, Tommasino M (2016). Prevalence and concordance of human papillomavirus infection at multiple anatomic sites among HIV-infected women from Chennai, India. Int J STD AIDS.

[24] Zhang HY, Fei MD, Jiang Y (2014). The diversity of human papillomavirus infection among human immunodeficiency virus-infected women in Yunnan, China. Virol J.

[25] Mane A, Nirmalkar A, Risbud AR, Vermund SH, Mehendale SM, Sahasrabuddhe VV (2012). HPV genotype distribution in cervical intraepithelial neoplasia among HIV-infected women in Pune, India. PLoS One.

[26] Aggarwal R, Sachdeva RK, Naru J, Suri V, Sharma A, Nijhawan R (2012). HPV genotyping in north Indian women infected with HIV. Int J Gynecol Pathol.

[27] Rocha-Brischiliari SC, Gimenes F, de Abreu AL (2014). Risk factors for cervical HPV infection and genotypes distribution in HIV-infected South Brazilian women. Infect Agent Cancer.

[28] Dames DN, Blackman E, Butler R (2014). High-risk cervical human papillomavirus infections among human immunodeficiency virus-positive women in the Bahamas. PLoS One.

[29] Grinsztejn B, Veloso VG, Levi JE (2009). Factors associated with increased prevalence of human papillomavirus infection in a cohort of HIV-infected Brazilian women. Int J Infect Dis.

[30] Konopnicki D, Manigart Y, Gilles C (2013). Sustained viral suppression and higher CD4+ T-cell count reduces the risk of persistent cervical high-risk human papillomavirus infection in HIV-positive women. J Infect Dis.

[31] Blitz S, Baxter J, Raboud J (2013). Evaluation of HIV and highly active antiretroviral therapy on the natural history of human papillomavirus infection and cervical cytopathologic findings in HIV-positive and high-risk HIV-negative women. J Infect Dis.

[32] Memiah P, Makokha V, Mbuthia W (2015). Epidemiology of cervical squamous intraepithelial lesions in HIV infected women in Kenya: a cross-sectional study. Afr J Reprod Health.

[33] Huchko MJ, Leslie H, Sneden J (2014). Risk factors for cervical precancer detection among previously unscreened HIV-infected women in Western Kenya. Int J Cancer.

[34] Mabeya H, Khozaim K, Liu T (2012). Comparison of conventional cervical cytology versus visual inspection with acetic acid among human immunodeficiency virus-infected women in Western Kenya. J Low Genit Tract Dis.

[35] Ezechi OC, Pettersson KO, Okolo CA, Ujah IA, Ostergren PO (2014). The association between HIV infection, antiretroviral therapy and cervical squamous intraepithelial lesions in South Western Nigerian women. PLoS One.

[36] Firnhaber C, Van Le H, Pettifor A (2010). Association between cervical dysplasia and human papillomavirus in HIV seropositive women from Johannesburg South Africa. Cancer Causes Control.

[37] Mogtomo ML, Malieugoue LC, Djiepgang C, Wankam M, Moune A, Ngane AN (2009). Incidence of cervical disease associated to HPV in human immunodeficiency infected women under highly active antiretroviral therapy. Infect Agent Cancer.

[38] Sahasrabuddhe VV, Bhosale RA, Joshi SN (2010). Prevalence and predictors of colposcopic-histopathologically confirmed cervical intraepithelial neoplasia in HIV-infected women in India. PLoS One.

[39] de Andrade AC, Luz PM, Velasque L (2011). Factors associated with colposcopy-histopathology confirmed cervical intraepithelial neoplasia among HIV-infected women from Rio De Janeiro, Brazil. PLoS One.

[40] Patrelli TS, Gizzo S, Peri F (2013). Impact of highly active antiretroviral therapy on the natural history of cervical precancerous lesions: a 17-year institutional longitudinal cohort study. Reprod Sci.

[41] Kitchener H, Nelson L, Adams J (2007). Colposcopy is not necessary to assess the risk to the cervix in HIV-positive women: an international cohort study of cervical pathology in HIV-1 positive women. Int J Cancer.

[42] Chakravarty J, Chourasia A, Thakur M, Singh AK, Sundar S, Agrawal NR (2016). Prevalence of human papillomavirus infection & cervical abnormalities in HIV-positive women in eastern India. Indian J Med Res.

[43] Katumba AC, Reji E, Gitau T (2016). World Health Organisation staging, adherence to HAART and abnormal cervical smears amongst HIV-infected women attending a government hospital in Johannesburg, South Africa. S Afr J Infect Dis.

[44] Liu E, McCree R, Mtisi E (2016). Prevalence and risk factors of cervical squamous intraepithelial lesions among HIV-infected women in Dar es Salaam, Tanzania. Int J STD AIDS.

[45] Prabha Devi K, Bindhu Priya N (2013). Conventional pap smear screening in HIV seropositive women in South India. J Obstet Gynaecol India.

[46] Marchetti G, Comi L, Bini T (2013). HPV Infection in a cohort of HIV-positive men and women: prevalence of oncogenic genotypes and predictors of mucosal damage at genital and oral sites. J Sex Transm Dis.

[47] Chalermchockcharoenkit A, Chayachinda C, Thamkhantho M, Komoltri C (2011). Prevalence and cumulative incidence of abnormal cervical cytology among HIV-infected Thai women: a 5·5-year retrospective cohort study. BMC Infect Dis.

[48] Drogoul-Vey MP, Marimoutou C, Robaglia-Schlupp A (2007). Determinants and evolution of squamous intraepithelial lesions in HIV-infected women, 1991–2004. AIDS Care.

[49] Mangclaviraj S, Kerr SJ, Chaithongwongwatthana S (2008). Nadir CD4 count and monthly income predict cervical squamous cell abnormalities in HIV-positive women in a resource-limited setting. Int J STD AIDS.

[50] Jaquet A, Horo A, Ekouevi DK (2014). Risk factors for cervical intraepithelial neoplasia in HIV-infected women on antiretroviral treatment in Cote d'Ivoire, West Africa. PLoS One.

[51] Oliveira PM, Oliveira RPC, Travessa IEM, Gomes MVC, dos Santos MLJ, Grassi MFR (2010). Prevalence and risk factors for cervical intraepithelial neoplasia in HIV-infected women in Salvador, Bahia, Brazil. Sao Paulo Med J.

[52] Gedefaw A, Astatkie A, Tessema GA (2013). The prevalence of precancerous cervical cancer lesion among HIV-infected women in southern Ethiopia: a cross-sectional study. PLoS One.

[53] Curry CL, Sage YH, Vragovic O, Stier EA (2012). Minimally abnormal pap testing and cervical histology in hiv-infected women. J Womens Health.

[54] Adler DH, Kakinami L, Modisenyane T (2012). Increased regression and decreased incidence of human papillomavirus-related cervical lesions among HIV-infected women on HAART. AIDS.

[55] Firnhaber C, Westreich D, Schulze D (2012). Highly active antiretroviral therapy and cervical dysplasia in HIV-positive women in South Africa. J Int AIDS Soc.

[56] Kreitchmann R, Bajotto H, da Silva DA, Fuchs SC (2013). Squamous intraepithelial lesions in HIV-infected women: prevalence, incidence, progression and regression. Arch Gynecol Obstet.

[57] Sirera G, Videla S, Lopez-Blazquez R (2008). Highly active antiretroviral therapy and incidence of cervical squamous intraepithelial lesions among HIV-infected women with normal cytology and CD4 counts above 350 cells/mm^3^. J Antimicrob Chemother.

[58] Soncini E, Zoncada A, Condemi V, Antoni AD, Bocchialini E, Soregotti P (2007). Reduction of the risk of cervical intraepithelial neoplasia in HIV-infected women treated with highly active antiretroviral therapy. Acta Biomed.

[59] Lehtovirta P, Finne P, Nieminen P (2006). Prevalence and risk factors of squamous intraepithelial lesions of the cervix among HIV-infected women—a long-term follow-up study in a low-prevalence population. Int J STD AIDS.

[60] Heard I, Potard V, Costagliola D (2006). Limited impact of immunosuppression and HAART on the incidence of cervical squamous intraepithelial lesions in HIV-positive women. Antiviral Therapy.

[61] Schuman P, Ohmit SE, Klein RS (2003). Longitudinal study of cervical squamous intraepithelial lesions in human immunodeficiency virus (HIV)-seropositive and at-risk HIV-seronegative women. J Infect Dis.

[62] Ellerbrock TV, Chiasson MA, Bush TJ (2000). Incidence of cervical squamous intraepithelial lesions in HIV-infected women. JAMA.

[63] Clifford GM, Franceschi S, Keiser O (2016). Immunodeficiency and the risk of cervical intraepithelial neoplasia 2/3 and cervical cancer: A nested case-control study in the Swiss HIV cohort study. Int J Cancer.

[64] Zeier MD, Botha MH, van der Merwe FH (2012). Progression and persistence of low-grade cervical squamous intraepithelial lesions in women living with human immunodeficiency virus. J Low Genit Tract Dis.

[65] Omar T, Schwartz S, Hanrahan C (2011). Progression and regression of premalignant cervical lesions in HIV-infected women from Soweto: a prospective cohort. AIDS.

[66] Kim SC, Messing S, Shah K, Luque AE (2013). Effect of highly active antiretroviral therapy (HAART) and menopause on risk of progression of cervical dysplasia in human immune-deficiency virus- (HIV-) infected women. Infect Dis Obstet Gynecol.

[67] Paramsothy P, Jamieson DJ, Heilig CM (2009). The effect of highly active antiretroviral therapy on human papillomavirus clearance and cervical cytology. Obstet Gynecol.

[68] Minkoff H, Ahdieh L, Massad LS (2001). The effect of highly active antiretroviral therapy on cervical cytologic changes associated with oncogenic HPV among HIV-infected women. AIDS.

[69] Lillo FB, Ferrari D, Veglia F (2001). Human papillomavirus infection and associated cervical disease in human immunodeficiency virus-infected women: effect of highly active antiretroviral therapy. J Infect Dis.

[70] Massad LS, Evans CT, Minkoff H (2004). Natural history of grade 1 cervical intraepithelial neoplasia in women with human immunodeficiency virus. Obstet Gynecol.

[71] Heard I, Tassie JM, Kazatchkine MD, Orth G (2002). Highly active antiretroviral therapy enhances regression of cervical intraepithelial neoplasia in HIV-seropositive women. AIDS.

[72] Del Mistro A, Bertorelle R, Franzetti M (2004). Antiretroviral therapy and the clinical evolution of human papillomavirus-associated genital lesions in HIV-positive women. Clin Infect Dis.

[73] Chen YC, Li CY, Liu HY, Lee NY, Ko WC, Ko NY (2014). Effect of antiretroviral therapy on the incidence of cervical neoplasia among HIV-infected women: a population-based cohort study in Taiwan. AIDS.

[74] Guiguet M, Boue F, Cadranel J, Lang JM, Rosenthal E, Costagliola D (2009). Effect of immunodeficiency, HIV viral load, and antiretroviral therapy on the risk of individual malignancies (FHDH-ANRS CO4): a prospective cohort study. Lancet Oncol.

[75] Cobucci RN, Lima PH, de Souza PC (2015). Assessing the impact of HAART on the incidence of defining and non-defining AIDS cancers among patients with HIV/AIDS: a systematic review. J Infect Public Health.

