# Supplementary material for: Association of antiretroviral therapy with high-risk human papillomavirus, cervical intraepithelial neoplasia, and invasive cervical cancer in women living with HIV: a systematic review and meta-analysis
Source: Lancet HIV. 2017 Oct 26;5(1):e45–58. doi: 10.1016/S2352-3018(17)30149-2 (PMC5757426; doi:10.1016/S2352-3018(17)30149-2)
Supplement: Supplementary appendix [file mmc1.pdf]

THE LANCET HIV

Supplementary appendix

This appendix formed part of the original submission and has been peer reviewed. We post it as supplied by the authors.

Supplement to: Kelly H, Weiss HA, Benavente Y, et al. Association of antiretroviral therapy with high-risk human papillomavirus, cervical intraepithelial neoplasia, and invasive cervical cancer in women living with HIV: a systematic review and meta-analysis. *Lancet HIV* 2017; published online Oct 26. http://dx.doi.org/10.1016/S2352-3018(17)30149-2.

Antiretroviral therapy, high-risk human papillomavirus and cervical intraepithelial neoplasia: a systematic review and meta-analysis

Supplementary information

The search strategy in MEDLINE and EMBASE on 6 May 2017

HPV STUDIES:

HPV [Title/Abstract] OR Human papillomavirus[Title/Abstract] AND Antiretroviral therapy [Title/Abstract] OR ART [Title/Abstract] OR Antiretroviral therapy, highly active [MeSH Terms] AND Cervix Uteri [MeSH Terms] OR Uterine cervix [MeSH Terms] OR cervical

CERVICAL LESION STUDIES:

Intraepithelial neoplasia [Title/Abstract] OR Squamous intraepithelial neoplasia [Title/Abstract] OR Neoplasms [MeSH Terms] OR Precancer [MeSH Terms] OR Cancer [Title/Abstract] OR Intraepithelial lesion [Title/Abstract] OR Carcinoma, Squamous Cell [MeSH Terms] OR Precursor lesions [Title/Abstract] OR Cervical intraepithelial lesion [MeSH Terms] AND Cervical [Title/Abstract] OR Cervix uteri [MeSH Terms] OR Uterine cervix [MeSH Terms] AND Antiretroviral therapy [Title/Abstract] OR ART [Title/Abstract] OR Antiretroviral therapy, highly active [MeSH Terms]

Supplementary Table 1. Summary of studies of the association of ART with HR-HPV prevalence

First author, year	Study design	Location	Sample size	HR-HPV+ prevalence	CD4+ count, cells per µl Median [IQR] or mean (SD or range)	Comparison group	Crude Odds Ratio (95% CI)	Adjusted Odds Ratio (95% CI)
Africa								
Kelly, 2017[1]	Cohort	Burkina Faso- Ouagadougou	570	59.3%	ART-naïve: 417 (315-606)<br>ART: 446 (309-600)	ART vs. ART-naive	0.95 (0.66-1.39)	0.76 (0.47-1.22)[a]
Kelly, 2017[1]	Cohort	South Africa-Johannesburg	613	78.8%	ART-naïve: 448 (353-614)<br>ART: 420 (279-567)	ART vs. ART-naive	0.69 (0.45-1.05)	0.61 (0.37-1.01)[b]
Zeier, 2015[2]	Cohort: ART initiation	South Africa –Western Cape	300	94.3%	(Mean, SD):<br>[3]194 [77-311]	ART vs. 'not on treatment', i.e. not yet initiated or interruption for >1 month	0.33 (0.24-0.44)	0.72 (0.39-1.32)[c]
Ezechi, 2014 [4]	Cross-sectional	Nigeria-Ogun & Lagos	220	24.5%	500 [347-685]	ART vs. ART-naive	0.36 (0.19-0.69)	0.40 (0.30-0.50)[d]
Reddy, 2014 [5]	Cross-sectional	Malawi-Lilongwe	294	38.8%	337 [215,491]	ART vs. ART-naive	0.78 (0.40-1.52)	0.71 (0.30-1.67)[e]
Rositch, 2013 [6]	Cohort: ART initiation	Uganda-Rakai	96	71.9%	216 (pre-ART)	6 months after vs. before ART-initiation	1.16 (0.62-2.16)	-
De Vuyst, 2012 [7]	Cross-sectional	Kenya-Nairobi	497	52.7%	ART naïve: 407<br>In ART users <2y: 333<br>In ART users ≥2y: 483	cART vs. cART-naive	0.75 (0.49-1.13)	0.64 (0.40-1.02)[f]
Jaquet, 2012 [8]	Cross-sectional	Côte d'Ivoire-Abidjan	254	52.8%	471 [318-629]	ART vs. ART-naive	1.07 (0.60-1.88)	0.84 (0.46 - 1.54)[g]
Veldhuijzen, 2011 [9]	Cross-sectional	Rwanda-Kigali	124	50.8%		ART vs. ART-naive	0.77 (0.38-1.59)	-
Asia								
Menezes, 2015 [10]	Cross-sectional	India-Chennai	50	48.0%	425 [range: 106-1229]	ART vs. ART-naive	0.61 (0.20-1.88)	-
Zhang, 2014 [11]	Cross-sectional	China-Yunnan	301	37.5%	571	ART vs. ART-naive	0.97 (0.60-1.58)	2.30 (1.09-4.85)[h]
Mane, 2012 [3]	Cross-sectional	India-Pune	277	35.3%	372 [241-556]	ART vs. ART-naive	-	1.46 (0.84-2.54)[i]
Aggarwal, 2012 [12]	Cross-sectional	India-Chandigarh	130	20.0%	398	HAART vs. HAART-naive	3.11 (0.87-11.13)	-
Latin America								
Rocha-Brischiliari, 2014 [13]	Cross-sectional	Brazil-Maringa city	178	46.6%	64% with CD4+≥200	HAART vs. HAART-naive	1.04 (0.50-2.14)	-
Dames, 2014 [14]	Cross-sectional	Bahamas-Nassau	165	78.2%	47% with CD4+ >200	HAART vs. HAART-naive	1.06 (0.41-2.70)	
Grinsztejn, 2009 [15]	Cross-sectional	Brazil-Rio de Janeiro	634	45.0%	74% with CD4+≥200	HAART ≥2 months vs. HAART-naive	-	1.09 (0.82-1.44)[j]
Europe/North America								
Konopnicki, 2013 [16]	Cohort	Belgium-Brussels	652	42.8%	426 [302-601]	cART vs. cART-naive	0.73 (0.50-1.07)	0.72 (0.41-1.27)[k]
Blitz, 2013 [17]	Cohort	Canada-11 cities	750	46.3%	336 [180-515]	HAART vs. ART-naïve or non-HAART regimen	0.70 (0.48-1.01)	-
Minkoff, 2010 [18]	Cohort: ART initiation	USA-5 US cities	286	22.4%	73% with CD4+ ≥200	Adherent ART users 30 months after vs. before ART initiation (within woman analysis)	-	0.60 (0.44-0.81)[l]
Fife, 2009 [19]	Cohort: ART initiation	USA/Puerto Rico	146	62.0%	238 [121-339]	6 months after vs. before ART-initiation (within woman analysis)	0.40 (0.24-0.69)	0.83 (0.74-0.94)[m]

Crude Odds Ratio (OR)=unadjusted OR; NR=not reported; cART=combination ART; HAART=highly active antiretroviral therapy; [a]adjusted Odds Ratio, adjusted for **CD4+ count, ART duration,** alcohol use, marital status, age at first pregnancy and cervicitis [re-analysis of published data],**;** [b]adjusted Odds Ratio, adjusted for **CD4+ count, ART duration**, age, smoking, injectable contraception, genital warts, condom use, vaginal cleansing, *Chlamydia trachomatis*, Bacterial vaginosis and *Trichomonas vaginalis*[re-analysis of published data]**;** [c]adjusted Odds Ratio, adjusted for **CD4+ count, ART duration**, age, sexual activity, months since cervical lesion excision, HIV-1 plasma and cervical viral load; [d]adjusted Odds Ratio, adjusted for age, type of community, life time sexual partner and marital status; [e]adjusted for **nadir CD4,**; months since HIV diagnosis and age [re-analysis of published data]; [f]adjusted Odds Ratio, adjusted for **CD4+ count, ART duration** and age [re-analysis of published data]; [g]adjusted for **CD4+ count**, age, marital status and age at first sex[re-analysis of published data]**;** [h]adjusted Odds Ratio, adjusted for **CD4+ count, ART duration** and age[re-analysis of published data]; [i]Adjusted Odds Ratio, adjusted for **CD4+ count**, age, marital status, education, income, parity, age at first sex, lifetime sex partners, past history of sexually transmitted infections and tobacco**;** [j]Adjusted Prevalence Ratio, adjusted for **nadir CD4+ count,** age, marital status, drug use, age at first sex, lifetime sex partners, history of HPV infection and condom use; [k]Adjusted Odds Ratio, adjusted for **current and nadir CD4+ count**, **ART duration**, age, CDC stage, duration of HIV and HIV plasma viral load; [l]Adjusted Odds Ratio, adjusted for **CD4+ count pre- and post-HAART** and treatment of CIN (age, lifetime sex partners, smoking, ethnicity had no impact on findings); [m]adjusted Odds Ratio, adjusted for **baseline CD4+** count (nadir as women initiated ART at baseline**)**, age, sexual activity at baseline, low-grade squamous intraepithelial lesions or higher at baseline and HIV-1 plasma viral load.

Supplementary Table 2. Summary of studies of the association of ART with high-grade cervical lesion prevalence

First author, year	Study design	Location	Sample size	CD4+ count, cells per µl Median [IQR] or mean (SD or range)	Lesion definition	Lesion prevalence	Comparison group	Crude Odds Ratio (95% CI)	Adjusted Odds Ratio (95% CI)
Africa									
Kelly, 2017[1]	Cohort	Burkina Faso-Ouagadougou	530	ART-naïve: 417 (315-606)<br>ART: 446 (309-600)	CIN2+	5.8%	ART vs. ART-naive	1.63 (0.66-4.04)	0.86 (0.26-2.83)[a]
Kelly, 2017[1]	Cohort	South Africa-Johannesburg	613	ART-naïve: 448 (353-614)<br>ART: 420 (279-567)	CIN2+	22.4%	ART vs. ART-naive	0.80 (0.53-1.20)	0.54 (0.32-0.91)[b]
Memiah, 2015 [20]	Cross-sectional	Kenya-Kiambu	686	45% had baseline CD4+ <200 cells/mm3	CIN2+	6.1%	ART vs. ART-pre-ART	0.55 (0.19-1.56)	-
Huchko, 2014 [21]	Cross-sectional	Kenya-Kisumu	3185	356 [218-530]	CIN2+	9.0%	HAART vs. HAART-naïve	0.96 (0.75-1.23)	1.01 (0.79-1.30)[c]
De Vuyst, 2012 [7]	Cross-sectional	Kenya-Nairobi	470	ART naïve: 407<br>ART users <2 yrs: 333<br>ART users ≥2 yrs: 483	CIN2+	24.0%	cART vs. cART-naïve	1.12 (0.69-1.84)	0.91 (0.51-1.62)[d]
Mabeya, 2012 [22]	Cross-sectional	Kenya-Eldoret	149	Mean (range): 400 (10-1198)	CIN2+	30.9%	HAART vs. HAART-naïve	1.18 (0.56-2.49)	-
Ezechi, 2014 [23]	Cross-sectional	Nigeria-Ogun & Lagos	490	mean: 532 [263-801]	HSIL+	5.1%	ART vs. ART-naive	0.44 (0.19-1.01)	-
Firnhaber, 2010 [24]	Cross-sectional	South Africa-Johannesburg	1010	231 (range:1-1789)	HSIL+	18.0%	HAART vs. HAART-naïve	1.31 (0.92-1.85)	-
Mogtomo, 2009 [25]	Cross-sectional	Cameroon-Douala	70	Mean : 253 among ART and 165 among ART-naive	HSIL+	31.4%	HAART vs. HAART-naïve	0.44 (0.16-1.26)	-
Asia									
Feng, 2012 [26]	Cross-sectional	China-Yunnan	301	571	CIN2+	9.3%	HAART vs. HAART-naïve	0.45 (0.20-0.98)	0.14 (0.02-1.09)[e]
Sahasrabuddhe, 2010 [27]	Cross-sectional	India-Pune	271	343 [244-495]	CIN2+	15.9%	ART vs. ART-naïve	2.16 (1.09-4.28) [f]	-
Latin America									
De Andrade, 2011 [28]	Cohort	Brazil-Rio de Janeiro	340	347 [193-546]	CIN2+	6.5%	ART (≥2 months) vs. ART-naive	2.31 (1.02-5.22)	-
Europe									
Patrelli, 2013 [29]	Cohort	Italy-Parma	194	ART-naïve: 487 [±238]<br>ART users: 411 [±188]	HSIL+	35.1%	Receiving ART vs. ART-naïve or women refusing treatment (but in need of treatment)	0.64 (0.35-1.18)	-
Kitchener, 2007[30]	Cohort	Europe-6 cities*	1026	27% women <200	HSIL+	10.0%	HAART vs. ART-naïve/other	1.12 (0.63-1.98)	1.26 (0.51-3.11)[g]

Crude Odds Ratio (OR)=unadjusted OR; NR=not reported; cART=combination ART; HAART=highly active antiretroviral therapy; [a]adjusted for **CD4+ count**, **ART duration,** age, Bacterial vaginosis and cervical ectopy[re-analysis of published data]; [b]adjusted for **CD4+ count**, **ART duration,** age at first pregnancy, injectable contraception and number of lifetime sex partners[re-analysis of published data]; [c]Adjusted for age and site; [d]Adjusted for **CD4+ count**, **ART duration** and age[re-analysis of published data]; [e]Adjusted for **CD4+ count**, **ART duration** and age[re-analysis of published data]; [f]Unadjusted; [g]Rate Ratio adjusted for baseline cytology, colposcopy, HPV and smoking. *includes women enrolled in Dublin- Ireland; Edinburgh and London- UK; Milan-Italy; Paris-France; and Warsaw-Poland

Supplementary Table 3. Summary of studies reporting the association of ART with cervical lesion outcomes and invasive cervical cancer incidence

First author, year	Location	Sample size	CD4+ count, cells per µl Median [IQR] or mean (SD)	Median interval between smears (months)	Outcome definition	Comparison group	Effect estimate (ES)	Adjusted ES (95%CI)
**Incidence studies**								
Africa								
Adler, 2012 [31]	South Africa-Soweto	767	CD4+ count <350; ART users: 56% ART naïve: 42%	14 [range: 6-77]	Normal to ASCUS+	ART ≥6 mths vs. ART-naive at baseline	HR	0.62 (0.42-0.91)[a]
Firnhaber, 2012 [32]	South Africa-Johannesburg	326	ART users: 248 (152,382); ART-naive: 299 (174,448)	6	Normal to ASCUS+	ART at baseline vs. ART-naive at baseline	HR	0.55 (0.34-0.90)[b]
Kelly, 2017[1]	South Africa-Johannesburg	379	ART users: 439[322-604] ART-naïve: 437 [346-543]	16	<CIN2 to CIN2/3	ART >16 months vs. ART-naive	OR	0.39 (0.15-1.01)[c]
Latin America								
Kreitchmann, 2013 [33]	Brazil-Porto Alegre	349	436 [range: 9-1571]	14	<LSIL to LSIL+	HAART vs. HAART-naive	HR	1.90 (0.90-4.01)[d]
Europe/North America								
Minkoff, 2010 [18]	USA- 5 cities	286	73% with CD4+ ≥200	6	Normal to ASCUS+	Adherent ART period vs. pre-HAART period (within-woman analysis)	HR	0.68 (0.25-1.85)[e]
Sirera, 2008 [34]	Spain- Barcelona	127	Mean: ART users: 646 ART naive: 681	12	Normal to LSIL+	HAART prior to baseline vs. ART-naive throughout FU	HR	1.66 (0.16-16.85)[f]
Soncini, 2007 [35]	Italy-Parma	101	50% with CD4+ count between 200-499	6	Normal to LSIL+	HAART (time-dependent) vs. ART-naïve	HR	0.30 (0.13-0.68)[g]
Lehtovirta, 2006 [36]	Finland-Helsinki	55	45% with CD4+ >500	6	Normal to LSIL+	HAART during FU vs. ART-naive	HR	0.80 (0.35-1.83)[h]
Heard, 2006 [37]	France- Paris	298	400 [250,574]	6	Normal to ASCUS+	HAART during FU vs. non-HAART and ART-naive	HR	0.70 (0.40-1.20)[i]
Schuman, 2003 [38]	USA- 4 cities	629	16% with CD4+ <200; not stratified by ART	6	Normal to LSIL+	HAART (time-dependent) vs. ART-naive	HR	1.20 (0.49-2.94)[j]
Ellerbrock, 2000 [39]	USA –New York	328	429; 24% with CD4+ <200	6	Normal to ASCUS+	ARV (time-dependent) vs. ARV-naive	HR	1.00 (0.50-2.00)[k]
Clifford, 2016 [40]	Switzerland-5 cities	1451	NR	~5 years	<CIN2 to CIN2/3	ART >2years vs. ART naive	OR	0.64 (0.42-0.98)[l]
**Progression studies**								
Africa								
Zeier, 2012 [41]	South Africa- Western Cape	1,048	312	Not reported	LSIL to HSIL+	HAART (starting before first LSIL detection) vs. naive	HR	0.66 (0.54-0.81)[m]
Firnhaber, 2012 [32]	South Africa -Johannesburg	326	As before	As before	Normal to LSIL+; LSIL to HSIL+	ART at baseline vs. ART-naive at baseline	HR	0.52 (0.27-1.01)[n]
Omar, 2011 [42]	South Africa- Soweto	1,074	356 [215,474]	5.5	Normal to LSIL+; LSIL to HSIL+/ASCH	HAART (time-varying) vs. naive	HR	0.72 (0.52-0.99)[o]
Adler, 2012 [31]	South Africa-Soweto	1,123	As before	As before	Subsequent smear with worsening dysplasia	ART ≥6 mths vs. ART-naive at baseline	OR	0.80 (0.57-1.13)[p]
Europe/North America								
Kim, 2013 [43]	USA- New York	245	Nadir: 206	12	normal->ASCUS+; ASCUS->LSIL+	HAART vs. other regimens or ART naïve	HR	0.47 (0.33-0.68)[q]
Blitz, 2013 [17]	Canada- 11 cities	326	336 [180, 515]	8 [6,13]	ASCUS to greater	HAART during study vs. ART-naïve or non-HAART	HR	1.02 (0.40-2.59)[r]
Paramsothy, 2009 [44]	USA- 4 cities	537	ART-naïve: 48% CD4≥500 vs. 24% of ART users	6	Normal to ASCUS; ASCUS to LSIL; LSIL to HSIL	HAART use during study period vs. pre-HAART or never on ART	HR	0.70 (0.60-1.00)[s]
Schuman, 2003 [38]	USA- 4 cities	629	As before	As before	Normal/ASCUS to LSIL+, LSIL to HSIL	HAART (time-dependent) vs. ART-naive	OR	1.50 (0.90-2.49)[t]
Minkoff, 2001 [45]	USA- 6 cities	741	36% with CD4<200	6	subsequent pap with any grade higher than baseline	HAART vs. off-HAART (women never received HAART or among initiators, prior to HAART initiation	OR	0.68 (0.52-0.88)[u]
Lillo, 2001 [46]	Italy- Milan	163	HAART : 260 (±23); ART-naïve: 627 (±38)	6	Normal to LSIL+; LSIL to HSIL	HAART during study vs. ART-naïve or non-HAART	OR	3.50 (1.01-12.12)[v]

First author, year	Location	Sample size	CD4+ count, cells per µl Median [IQR] or mean (SD)	Median interval between smears (months)	Outcome definition	Comparison group	Effect estimate (ES)	Adjusted ES (95%CI)
**Regression studies**								
Africa								
Zeier, 2012 [41]	South Africa- Western Cape	1,048	312	Not reported	≥LSIL to <LSIL (2 normal results at least 4 weeks apart)	HAART (starting before first LSIL detection) vs. naive	HR	1.71 (1.29-2.27)[m]
Adler, 2012 [31]	South Africa-Soweto	1,123	As before	As before	Subsequent improvement in cytological results	ART ≥6 mths vs. ART-naive at baseline	OR	2.61 (1.75-3.89) [p]
Europe/North America								
Blitz, 2013 [17]	Canada- 11 cities	326	As before	As before	≥ASCUS to <ASCUS	HAART during study vs. ART-naïve or non-HAART	HR	3.32 (1.22-9.04)[r]
Minkoff, 2010 [18]	USA-5 US cities	286	As before	As before	SIL to lower grade	Adherent ART period vs. pre-HAART period (within-woman analysis)	HR	2.25 (1.03-4.93)[w]
Paramsothy, 2009 [44]	USA- 4 cities	537	As before	As before	HSIL to LSIL, LSIL to ASCUS, ASCUS to normal	HAART use during study period vs. pre-HAART or never on ART	HR	1.30 (1.00-1.70)[s]
Massad, 2004[47]	USA-6 cities	202	259	6	Regression from CIN1 to normal vs. CIN persistence or progression to >CIN1	HAART at time of regression vs. HAART-naive	HR	1.32 (0.71-2.50)[x]
Heard, 2002 [48]	France- Paris	168	250 [139-400]	6	Reversion to normal or from HG to LG	HAART (time-dependent) vs. ART-naive	HR	1.93 (1.14-3.29)[y]
Del Mistro, 2004 [49]	Italy- Vicenza & Padova	201	292 (range: 2-1445)	6-12	LGSIL or HGSIL to Normal or lower SIL grade at subsequent exam	HAART at baseline and FU vs. ART-naive at baseline and FU OR non-HAART OR change in regimen during FU	OR	1.87 (0.71-4.93)[r]
Schuman, 2003 [38]	USA- 4 cities	629	As before	As before	≥LSIL+ to <LSIL	HAART (time-dependent) vs. ART-naive	OR	0.86 (0.50-1.47) [t]
Minkoff, 2001 [45]	USA- 6 cities	741	As before	As before	Lower grade abnormality than baseline	HAART vs. off-HAART (women never received HAART or among initiators, prior to HAART initiation	OR	1.40 (1.04-1.82) [u]
**Invasive cervical cancer incidence studies**								
Clifford, 2016 [40]	Switzerland-5 cities	80	Stable among ART users over time, but decreasing among ART-naïve	Median ~5 years	<CIN2 to ICC	ART >2years vs. ART naive	OR	0.34 (0.05-2.26)[l]
Chen, 2014[50]	Taiwan	1360	NR	3.2 years	Incidence of CIS or ICC	ART for >6 months vs. ART-naive or ART for ≤6 months	HR	0.20 (0.05-0.77)[z]
Guiguet, 2009[51]	France -62 French university hospitals	14,406	Nadir: 158 [83-253]; Time of ICC diagnosis: ART users=307 [167-474] ART-naïve=267 [157-401]	Median 5.0 years [IQR: 2.2-7.7)	Incidence of ICC	ART for ≥6 months vs. ART-naive, dual therapy, or cART for <6 months	RR	0.50 (0.30-0.90)[yz]

HR=Hazard Ratio; OR=Odd Ratio; RR=Rate Ratio; CIS-cervical cancer *in situ*; ICC=invasive cervical cancer; NR-not reported; FU=follow-up; [a]adjusted for **time-varying ART**, current CD4+, body mass index, sexual activity, STI symptom, smoking; [b]adjusted for age, CD4 count, age at first intercourse, lifetime number of sexual partners, history of sexual transmitted diseases, use of hormonal contraception, condom use at last sex, employment status, current smoking, snuff use and education level; [c]adjusted for number of lifetime sex partners, **baseline CD4+ count and time on ART**; [d]age, education, log viral load and CD4+ count; [e]adjusted for **time-varying ART** and adherence; treatment of CIN (time-dependent), baseline CD4+ (age, lifetime sex partners, smoking, ethnicity had no impact on findings); [f]adjusted for CD4+ count (unclear if nadir or current); [g]adjusted for **time-varying ART** and baseline CD4+ count; [h]adjusted for age; [i]adjusted for **time-varying ART**, age, ethnicity, smoking, LTSP, contraception use, condom use; [j]adjusted for baseline CD4+, time (visit), study site, age, race/ethnicity and education contraception use, condom, inclusion period; [k]adjusted for **time-varying ART**, baseline CD4+, age, smoking, HPV persistence; [l]case-control study matched on enrolment centre, HIV-transmission category, age at enrolment, year of enrolment and adjusted for nadir CD4+, enrolment from university hospitals (Basel, Bern, Geneva, Lausanne and Zurich) and two cantonal hospitals (Gallen and Ticino); [m]adjusted for **duration on ART**, age and excision treatment; [n]adjusted for CD4+ at baseline; [o]adjusted for **time-varying ART**; CD4+ at baseline, age, baseline smear result, smoking; [p]adjusted for current CD4+, BMI, sexual activity, STI symptom, smoking; [q]adjusted for **time-varying CD4+,** duration of HIV infection, menopausal, HIV acquired through drug use; smoking; [r]unadjusted; [s]adjusted for **time-varying CD4+, time-varying ART,** HPV positive, baseline pap result; [t]adjusted for baseline CD4+, time (visit), study site, age, race/ethnicity and education; [u]adjusted for baseline CD4+ and baseline pap result; [v]adjusted for CD4+, HIV-1 RNA and gynecological treatment; [w]adjusted for **time-varying ART** and adherence; treatment of CIN (time-dependent), baseline CD4+ (age, lifetime sex partners, smoking, ethnicity had no impact on findings); [x]adjusted for time-varying ART, CD4+ count, HIV-PVL, current smoking, HR-HPV infection, ethnicity and age; [y]adjusted for **time-varying ART**, timing of CIN detection, CIN grade, CD4+ at lesion detection; [z]adjusted for age at HIV diagnosis, income, urbanization level, occupation, drug dependence, treated opportunistic infections, history of STI, frequency of pap test after HIV diagnosis; [yz]adjusted for time-varying ART, time-varying CD4+ count, time-varying age, sub-Saharan African origin.

Supplementary Table 4. Quality assessment of studies reporting the effect of ART on HR-HPV prevalence, and other significant findings reported

First author, year	Participant selection and Loss to follow-up [LTFU] (for cohort studies)	HR-HPV test method	Adjustment for confounding	Other significant published findings related to HIV	
				Comparison groups	Effect estimate, adjustment
Africa					
Kelly, 2017	WLHIV attending HIV outpatient and treatment centres in Ouagadougou and Johannesburg invited to participate in a study comparing cervical cancer screening methods. **Low risk**	INNO-LiPA HPV Genotyping Extra II. Quality Control (QC) of testing performed. **Low risk**	In BF: alcohol, marital status, age at first pregnancy, cervicitis, current CD4+ and ART duration. In SA: age, smoking, injectable contraception, genital warts, condom use, vaginal cleansing, *Chlamydia trachomatis*, BV, *Trichomonas vaginalis*, current CD4+ and ART duration. **Low risk**	Short duration ART (≤2yrs) vs. Long duration ART (≥2yrs)	BF: aPR=1.24 (95%CI:1.04-1.47), adjusted for CD4+
				CD4<200 vs. >500 cells/µl	BF: aPR=1.43 (95%CI:1.18-1.72), SA: aPR=1.15 (95%CI:1.01-1.30)
Zeier, 2015	Known HIV-positive women were approached for enrolment provided they were ART-naive. Decision to start ART based on South African guidelines. LTFU unclear. **Selection bias of ART initiators.**	Roche Linear Array. QC of testing performed. **Low risk**	Time dependent covariates: time on ART, HIV-1 PVL, age, sexual activity, months since excision. **Low risk**	Months since ART initiation (per months analysis)	aOR=0.95 (95%CI: 0.93-0.97), adjusted for time on ART, HIV-1 PVL, age, sexual activity, months since excision
Ezechi, 2014	80% recruited from cervical cancer screening clinic; 20% from community cervical cancer screening outreach in urban and rural locations.	Seegene Seeplex HPV4A ACE PC. Validated against Hybrid Capture 2[52]. **Low risk**	Age, type of community, LTSP, marital status; **no adjustment for CD4+ count or time on ART. High risk**	CD4 <200 vs. ≥500 cells/mm^3	aPR=2.40 (95%CI:1.70-5.90), adjusted for age, type of community, LTSP, marital status
Reddy, 2014	All women coming to clinic for routine ART or pre-ART care were given the opportunity to participate in the study. Large number on ART (82%) limits power to test association between ART and HR-HPV. **Medium risk**	PCR based (MY09/MY11). Not validated - High **risk**	Age, nadir CD4+ count, time since HIV diagnosis. Adjustment for other cofactors had no impact on findings (LTSP, AFS, history STI and circumcision status of partner). **Low risk**	-	-
Rositch, 2013	HIV/HSV-2 co-infected women enrolled in RCT of HSV-2 suppression to assess HIV disease progression; women who were ART-naïve and with CD4+ between 300-400 cells per µl were included; women initiated on ART if CD4+ <250 cells/µl. No LTFU. **Selection bias of ART initiators starting at low nadir CD4+ and short follow-up period (6 months). All women were HSV-2 infected (which is potential confounder for HPV).**	Linear Array using self-administered swabs. **Medium risk (**lower sensitivity for HPV detection using self-collection compared to clinician-collected)	Unadjusted analysis undertaken by current authors. **High risk** (Note: median nadir=216 cells/µl at ART initiation and women followed up to 6 months)	-	-
De Vuyst, 2012	WLHIV attending Coptic Hope Centre for Infectious Diseases for HIV related conditions invited to participate in a study comparing cervical cancer screening methods. **Low risk**	PCR based (GP5+/6+). Validated method. **Low risk**	Age, current CD4 count and ART duration. Adjustment for other cofactors had no impact on findings (marital status, LTSP, recent sex partners, HC use and number of pregnancies) **Low risk**	Long duration ART (≥2yrs) vs. ART-naive	aPR=0.77 (95%CI:0.61-0.96), adjusted for age
				CD4<250 vs. ≥500 cells/µl among ART-naive	aPR=1.51 (95%CI:1.02-2.25), adjusted for age
Jaquet, 2012	Women with no cervical neoplastic lesion (determined by visual inspection), consecutively enrolled, attending HIV clinic and recruited through cervical cancer screening programme in Abidjan. **Low risk**	Roche Linear Array. **Low risk**	Age, marital status, age at first sex, current CD4+. **Low risk**	CD4<200 vs. ≥500 cells/µl	aOR=2.8 (95%CI: 1.1-8.1), adjusted for age, marital status, age at first sex
Veldhuijzen, 2011	'High-risk' women testing HIV positive as part of a HIV prevalence survey in Kigali, Rwanda were invited to participate in a survey for HPV prevalence. Women recruited via community meetings in three districts, study conducted in an international non-governmental organisation. Low risk.	Roche Linear Array**. Low risk**	Unadjusted analysis undertaken by current authors. **High risk**	-	-

First author, year	Participant selection and Loss to follow-up [LTFU] (for cohort studies)	HR-HPV test method	Adjustment for confounding	Other significant published findings related to HIV	
				Comparison groups	Effect estimate, adjustment
Asia					
Menezes, 2015	Consecutive women receiving care at Y.R. Gaitonde Center for AIDS Education and Research in Chennai, India. Low sample number (n=50). **Medium risk**	PCR based (TS-E7-MPG). Validated against Hybrid Capture II and Linear Array [53]. **Low risk**	Unadjusted analysis due to small numbers. **High risk**	-	-
Zhang, 2014	Recruitment to HIV clinic based on linkages with VCT centres and referrals of known HIV infected women from provincial and prefecture level CDC affiliated ART clinics. Representative of HIV-positive women seeking cervical cancer screening services. **Low risk**	Digene Hybrid Capture II. **Low risk**	Adjusted for age, current CD4+ and ART duration. Adjustment for other cofactors had no impact on findings (AFS, LTSP, history STI, condom, parity). **Low risk**	-	-
Mane, 2012	Women attending outpatient gynaecology clinic in tertiary care hospital in Pune, India, recruited consecutively. **Low risk**	Roche Linear Array. QC of testing performed. **Low risk**	Age, marital status, education, family income, parity, AFS, LTSP, past STI, smoking, current CD4; Other typical confounding factors such as smoking, parity were not associated with HPV infection; **Low risk.**	-	-
Aggarwal, 2012	HIV-positive women randomly enrolled from ARV clinic. **Low risk**	Hybribio GenoArray. Validated against Roche Linear array [54]. **Low risk**	Unadjusted analysis undertaken by current authors. **High risk**	-	-
Latin America					
Rocha-Brischiliari, 2014	Women aged 18-66 years attending Specialized Assistance Service for STD/AIDS of Maringa city/Southern Brazil from April to Oct 2011. **Low risk.**	PCR based (MY09/MY11/HypCH4V) but not validated. **Unclear risk**	Unadjusted analysis undertaken by current authors. **High risk**	-	-
Dames, 2014	HIV sero-positive women ≥18yrs consecutively enrolled from Infectious Disease Clinic at the Princess Margaret Hospital in New Providence, Nassau, Bahamas Feb-Sep 2008. Clinic caters for all Bahaman islands, see 1500 HIV-positive each year but at time of this study, there was no cervical cancer screening programme (may explain high disease). **Low risk**	Hybrid Capture II+Linear Array). **Low risk**	Unadjusted analysis undertaken by current authors. **High risk**	CD4 ≤200 cells/µl vs. >200 cells/µl	aOR=7.27 (95%CI:1.41-37.53), adjusted for age, duration on ART, HIV PVL and cytological abnormality
Grinsztejn, 2009	Prospective open cohort (IPEC-Fiocruz) established in 1996, women followed up in a clinical research hospital in Rio de Janeiro. **Low risk**	Hybrid Capture II. **Low risk**	Age, marital status, drug use, age at first sex, LTSP, history of HPV infection, condom use, nadir CD4+ count. **Only Prevalence Ratio available. Medium risk**	Nadir CD4 <100 vs. ≥250 cells/ µl	aPR=1.56 (95%CI: 1.18-2.06), adjusted for age, marital status, drug use, age at first sex, LTSP, history of HPV, condom use

First author, year	Participant selection and Loss to follow-up [LTFU] (for cohort studies)	HR-HPV test method	Adjustment for confounding	Other significant published findings related to HIV	
				Comparison groups	Effect estimate, adjustment
Europe/North America					
Konopnicki, 2013	Women consecutively enrolled from cervical cancer screening program at AIDS reference centre in Saint-Pierre University Hospital from 2002 to 2010. Those who developed lesions during study period were censored. **84% of women were of SSA origin. Low risk**	Hybrid Capture II. **Low risk**	Age, current and nadir CD4+ count, CDC stage, duration of HIV follow-up, ART duration, HIV-1 viral suppression. Adjustment for other cofactors (previous pregnancy) had no impact on findings. **Low risk**	Long duration ART (≥2yrs) vs. short duration (<2yrs) or ART-naive	aPR=0.60 (95%CI:0.37-0.99), adjusted for age, current and nadir CD4+, CDC stage during HIV infection, ART status, HIV-1 viral suppression
				HIV VL suppression ≥2yrs vs. <2yrs	aPR=0.28 (95%CI:0.17-0.49), , adjusted for age, current and nadir CD4+, CDC stage during HIV infection, ART status, ART duration
				Nadir CD4 <500 vs. ≥500 cells/ µl	aPR=3.31 (95%CI:1.51-7.24), , adjusted for age, current CD4+, CDC stage during HIV infection, ART status, ART duration, HIV-1 viral suppression
				Per 100 CD4 cells/µl increase	aRR=0.89 (95%CI:0.85-0.93), , adjusted for age, current and nadir CD4+, CDC stage during HIV infection, ART status, ART duration, HIV-1 viral suppression
Blitz, 2013	HIV-positive women recruited from community-based or tertiary care centres. 71% of women attended >1 follow-up visit. **Medium risk -significant LTFU**	PCR based (MY09/MY11/HMB01 & PGMY). Validation unclear. **Low-medium risk**	Unadjusted analysis undertaken by current authors. **High risk**	-	-
Minkoff, 2010	Prospective follow-up of ART initiators in the Women's Interagency HIV Study (WIHS). **Selection bias of ART initiators.**	PCR based (MY09/MY11/HMB01). Validation unclear. **Low-medium risk**	Treatment of CIN, CD4+ count pre- and post-HAART. Women acted as their own comparator group. Minimises bias due to fact that women starting ART are sicker than those who do not yet need ART. Adjustment for other cofactors had no impact on findings (age, number sex partners in last 6 months, smoking, and race/ethnicity) **Low risk.**	-	-
Fife, 2009	Subjects enrolled when they were about to begin ART; either in controlled clinical trial or by prescription. 18% LTFU at 6 months and 36% loss to follow-up at 24 months. Hispanic subjects more likely to have HPV data at all time points. A higher rate of HR-HPV DNA detection at baseline associated (p=0.089) with missing HPV data for at least one visit. **Selection bias of ART initiators. High risk**	PCR based (Roche PCR/reverse blot strip assay). Validation unclear. **Low-medium risk**	Age, sexual activity at baseline and current, LSIL+ at baseline, CD4+ count, HIV-1 PVL. **Prevalence reported after compared to before - participants did not act as their own comparator group. Medium-High risk**	-	-

NR=not reported; aPR=adjusted Prevalence Ratio; PVL=plasma viral load; LTSP=lifetime sexual partners; AFS=age at first sexual intercourse; STI=sexually transmitted infection; HC=hormonal contraception; LSIL=low-grade squamous intraepithelial lesions

Supplementary Table 5. Quality assessment of studies reporting the effect of ART on high-grade cervical lesion prevalence, and other significant findings reported

First author, year	Participant selection	Adjustment for confounding	Endpoint determination & Biopsy decision	Other significant findings related to HIV	
				**Comparison groups**	**Effect estimate, adjustment factors**
Africa					
Kelly, 2016	WLHIV attending HIV outpatient and treatment centres in Ouagadougou and Johannesburg invited to participate in a study comparing cervical cancer screening methods. **Low risk**	In BF: age, BV, cervical ectopy, CD4+ and ART duration. Adjustment for other sexual behaviour cofactors had no impact on findings. In SA: age at first pregnancy, injectable contraception, LTSP, CD4+ and ART duration. **Low risk**	All participants were referred for colposcopy performed by trained colposcopists. Systematic 4-quadrant cervical biopsy, including directed biopsy of any suspicious lesions, was performed for participants who had abnormalities detected by cytology, VIA/VILI or colposcopy, or who were HR-HPV DNA positive (Digene HC-II). **Low risk**	Short duration ART (≤2yrs) vs. Long duration ART (≥2yrs)	SA: aOR=1.99 (95%CI:1.12-3.54), adjusted for CD4+
				ART-naive vs. Long duration ART (≥2yrs)	SA: aOR=1.87 (95%CI:1.11-3.17), adjusted for CD4+
Memiah, 2015	Consecutive enrolment of HIV positive women attending ART treatment clinic in Kiambu district, Kenya. Study site is a faith based hospital offering care and treatment to ~4000 HIV infected persons. **Medium risk**	Unadjusted analysis undertaken by current authors. **High risk**	Women positive on VILI were indicated for biopsy. No details on histology readings or any QA involved. **High risk.**	-	-
Huchko, 2014	Screening offered to women enrolled in care in two HIV clinics (Family AIDS Care and Education Services) in Kisumu, Kenya, no prior screening programmes were in place. **Low risk.**	Age and site. No adjustment for ART duration, HIV-1 PVL or CD4+. **High risk.**	Screening in 3 clinics; 814 (25%) women were referred for colposcopy if: abnormal VIA (main clinic); abnormal VIA and VILI (satellite clinic 1); and cytology result of ≥ASCUS+ (satellite clinic 2). Biopsies were taken when colposcopy abnormal or unsatisfactory (544 women (16.8% of all enrolled)). Histology results available for 15% of all enrolled women (n=488). **High risk - only 15% have histology result; 25% with colposcopy, remainder have combined VI/ cytology endpoint (possible underreporting of cervical disease)**	CD4. ≥500 vs. <200 among ART-naive	aOR=0.42 (95%CI:0.22-0.80), adjusted for hormonal contraception
				Nadir CD4<500 vs. <200 cells/µl	aOR=0.61 (95%CI:0.38-0.97), adjusted for months in HIV care and hormonal contraception
				Months on HAART	aOR=0.98 (95%CI: 0.95-1.01), adjusted for current CD4+, hormonal contraception
De Vuyst, 2012	HIV positive women attending Coptic Hope Centre for Infectious Diseases for HIV related conditions invited to participate in a study comparing cervical cancer screening methods. **Low risk**	Age, CD4+ and ART duration. **Low risk**	All women biopsied (from most abnormal area of cervix, or if no lesion was visualised at 12 o clock). Cytology slides and biopsies read by single pathologist at Aga Khan University of Nairobi. No information on QA. **All with histology result. Low risk**	CD4<250 vs. ≥500 among ART-naive	aPR=4.23 (95%CI:1.27-14.0), adjusted for age
				Long duration ART (≥2yrs) vs. ART-naive	aPR=0.88 (95%CI:0.57-1.35), adjusted for age
Mabeya, 2012	Women recruited from waiting rooms of HIV clinics at Moi University school of Medicine in Eldoret, Kenya. **Low risk**	Unadjusted analysis undertaken by authors. **High risk**	All participants underwent VIA and cytology pap smear done by nurse. A single punch biopsy taken from visible abnormal lesion with aid of colposcope; if no visible lesion, a single punch biopsy taken from either 6 or 12 o'clock. Blinding screening and reading. 10% of pap smears and biopsies read by an external pathologist. **All with histology result. Low risk**	-	-

First author, year	Participant selection	Adjustment for confounding	Endpoint determination & Biopsy decision	Other significant findings related to HIV	
				Comparison groups	Effect estimate, adjustment factors
Ezechi, 2014	Participants recruited at cervical cancer screening clinic, NIMR, Lagos and 10 communities of Lagos and Ogun States of Nigeria during community outreach programmes. Women who presented for cervical cancer screening at the NIMR clinic and during community outreach programmes were screened for eligibility for recruitment. **Low risk**	Unadjusted analysis undertaken by current authors. **High risk.** Original authors report the OR of HSIL+ vs Normal, and excludes those with ASCUS/LSIL from the analysis. Reported unadjusted analysis includes all women. **High risk (unadjusted)**	Cytology only. Interpretation of pap at Anapath laboratory using Bethesda system. Second reading by senior pathologist of all abnormal cases and 15% normal cases (n=358 [31%] in total). In event of discrepancy (5% -all ASCUS), slides sent to a second senior pathologist for independent review and final diagnosis attributed to this final review. Of 17 ASCUS cases, 1 was upgraded and 5 were downgraded. **Low risk.**	-	-
Firnhaber, 2010	Cross-sectional cohort recruitment from adult HIV outpatient clinic in teaching hospital affiliated with University of Witwatersrand. **Low risk**	Unadjusted OR undertaken by authors. **High risk**	Cytology only, read and analysed according to Bethesda system. 10% of cytology slides sent to University of North Carolina for blinded double reading on two occasions; high rate of concordance observed (81-85%). Of the 182 cases graded as HSIL+, 83 had pathology results; most HSIL cases were histologically confirmed as CIN2 (30%) or CIN3 (47%); 23% were classified as CIN1. **Low risk.**	CD4 <200 vs. ≥500	aPR=2.4 (95%CI:1.4-4.2), adjusted for age and ART
Mogtomo, 2009	Participants recruited in a day care centre at Bonassama hospital in Douala for HIV therapy. **Low risk**	Unadjusted analysis undertaken by current authors. **High risk**	Cytology only, read by single pathologist. Unclear whether QA was used. **High risk.**	-	-
Asia					
Feng, 2012	Recruitment through outreach to Women's and Children's Hospital of Luxi County in Mangshi, Dehong Perfecture by hospital personnel familiar to the local HIV-infected population. Unpublished data. **High risk**.	Age, CD4+ and ART duration. No adjustment for sex behaviour **Low-medium risk.**	Colposcopy performed on all. Biopsy performed on consenting participants with clinical evidence of cervical abnormalities (does not report N who had histology). Final diagnosis based on histology where biopsy was taken, and on colposcopic diagnosis where biopsy was not indicated or not taken. CIN2+ was 8.4% while HSIL+ was 1.1%. **High risk**	-	-
Sahasrabuddhe, 2010	Study participation offered to consecutive HIV-infected women in a public-sector ART centre in hospital premises. Participants also recruited through outreach efforts. No participant had previously been screened or treated for cervical abnormality. **Low risk**	Age, education, income, age at first sex, lifetime sex partners, parity, current CD4+, WHO stage of HIV disease, HR-HPV. No adjustment for ART duration or HIV-1 PVL. **High risk.**	Colposcopy performed on all. Biopsy (cervical punch, ECC or LEEP) performed on consenting participants with clinical evidence of cervical abnormalities (24.1%). Final diagnosis based on histology where biopsy was taken, and on colposcopic diagnosis where biopsy was not indicated or not taken. Colposcopy results served as final diagnosis for 81.2% (246/303) participants. Final histopathology results were available for 18.8% (57/303). QA of colposcopic images by a senior experienced gynaecologist. **High risk - only 19% have histology result**	-	-

First author, year	Participant selection	Adjustment for confounding	Endpoint determination & Biopsy decision	Other significant findings related to HIV	
				Comparison groups	Effect estimate, adjustment factors
Latin America					
De Andrade, 2011	Prospective open cohort at Evandro Chagas Clinical Research Institute, Oswaldo Cruz Foundation. **Low risk**	Unadjusted (reported by original authors). **High risk.**	Directed biopsies performed when minor colposcopic changes were observed but it was not possible to exclude CIN2+ and the pap test showed LSIL, ASC or normal. No information on how many had histology endpoint but authors report that all CIN2+/ICC were histopathologically reported. **High risk**	Nadir CD4<350 vs. ≥350 cells/µl	aPR=6.03 (95%CI:1.50-24.3), adjusted for Age, smoking, VIN and/or VAIN
Europe					
Patrellli, 2013	HIV-infected women admitted to the Department of Obstetrics and Gynaecology of the University of Parma, referred from Infectious Disease Clinic for early detection of HPV-related disease. No details on recruitment procedures or selection. **Medium risk**	Unadjusted analysis undertaken by current authors. **High risk**	Cytology by pap smear and colposcopy. Those with abnormal pap and colposcopy underwent targeted biopsy (48% of all women). All colposcopy performed by same examiner. Cytology and histology examined by gynaecological pathologists. No information on how many, or whether there was independent reading or QC. **Medium risk.** Authors mention bias could be attributed to period effect of cytology reporting over a 17 year period (frequency and reliability changed in subsequent years) and introduction of ASCUS class could have reduced number of high grade cases reported.	-	-
Kitchener, 2007	Women with HIV already under surveillance, or newly diagnosed recruited from 6 European cities between 2000 and 2003 (women recruited in South Africa were all ART-naïve and not include in the review/meta-analysis). Variability in ART use across cities (56-79%). **Low risk**.	Baseline cytology, colposcopy, HPV and smoking. No adjustment for CD4+ or sex behaviour. **High risk**.	Cytology performed every 6 months, read and analysed according to Bethesda system. Colposcopy and biopsy performed if clinically indicated.	-	-

NR=not reported; aOR=adjusted Odds Ratio; BV=bacterial vaginosis; LTSP=lifetime sexual partners; VIA/VILI=visual inspection using acetic acid or Lugol's iodine; HC-II=Hybrid Capture II; PVL=plasma viral load; ASCUS= atypical squamous cells of undetermined significance; LSIL=low-grade squamous intraepithelial lesions; ; HSIL=high-grade squamous intraepithelial lesions; QA=Quality Assurance; ECC-endocervical curettage; LEEP= Loop Electrosurgical Excision Procedure; VIN=vulvar intraepithelial neoplasia; VAIN=vaginal intraepithelial neoplasia

Supplementary Table 6. Quality assessment of studies reporting the effect of ART on cervical lesion outcomes and invasive cervical cancer incidence and other significant findings reported

First author, year	Participant selection, ART status and LTFU	Statistical methods used	Adjustment for confounding	Endpoint assessment	Other significant findings related to HIV	
					**(Outcome) Comparison groups**	**Effect estimate, adjustment**
Africa (in alphabetical order)						
Adler, 2012	Operational cohort of treatment-naive HIV infected women set up to transition patients onto ARV, receiving a package of care between 2003-2010. **Selection bias of ART initiators. 0% LTFU.**	For incidence: Survival analysis using generalised estimating equation, accounts for changes over time. **Low risk**<br>For progression/regression: marginal models which assumes no pattern in the correlation of observations within an individual; i.e. assess differences between individuals (a comparison of women on ART vs. women not on ART).<br><br>The survival analysis was restricted to the women not on HAART at baseline who had a normal baseline cervical smear result (n=767) and assessed the risk of progression to any abnormal result. Women started on HAART during the study period were included in the survival analysis after they had been receiving treatment for 180 days.	BMI, sex history, STI symptom, time since enrolment, current CD4+ count, time-varying CD4+ and ART. **Low risk**.	Cytology on all women analysed at NHLS which is accredited by South African National Accreditation System. All smears verified by second reader. **Low risk.**	(Incidence): Current CD4 ≤200 vs. >500	aHR=1.73 (95%CI:1.15-2.61), adjusted for BMI, LTSP, STI, smoking, ART
					(Progression): Current CD4 <200 vs. >500	aOR=2.50 (95%CI:1.67-3.73, adjusted for BMI, LTSP, STI, smoking, ART
Kelly, 2016	WLHIV attending HIV outpatient and treatment centres in Ouagadougou and Johannesburg invited to participate in a study comparing cervical cancer screening methods. 9% LTFU and a further 14% had missing/inadequate histology. **Low risk.**	Logistic regression. Not included in the meta-analysis.	Baseline CD4+ and lifetime number of sex partners. **Medium risk**	All participants were referred for colposcopy performed by trained colposcopists. Systematic 4-quadrant cervical biopsy, including directed biopsy of any suspicious lesions, was performed for participants who had abnormalities detected by cytology, VIA/VILI or colposcopy, or who were HR-HPV DNA positive (Digene HC-II). All histological slides from women with a local diagnosis of CIN2+ and approximately 10% of slides from women with ≤CIN1 histological findings were reviewed by the HARP Endpoint Committee of five pathologists, for consensus classification.<br>**Low risk**		-

First author, year	Participant selection, ART status and LTFU	Statistical methods used	Adjustment for confounding	Endpoint assessment	Other significant findings related to HIV	
					**(Outcome) Comparison groups**	**Effect estimate, adjustment**
Firnhaber, 2012	Observational longitudinal study included women 18-65yrs recruited from adult HIV outpatient clinic affiliated with teaching hospital in Johannesburg. 0% LTFU. **Low risk.**	For both incidence and progression: Poisson regression reporting Incidence Rate Ratio. Intent-to-treat analysis, so no adjustment for change in ART exposure after baseline were considered -**Low risk**	Age, CD4+ count, AFS, LTSP, history of STI, hormonal contraception, condom at last sex, employment, current smoking, snuff, education. No adjustment for change in ART exposure after baseline. **Medium risk**	Cytology on all; 10% of cytology slides sent to University of North Carolina for blinded double reading on two occasions; high rate of concordance observed (81-85%). Of the 182 cases graded as HSIL+, 83 had pathology results; most HSIL cases were histologically confirmed as CIN2 (30%) or CIN3 (47%); 23% were classified as CIN1. **Low risk.**	n/a	n/a
Omar, 2011	Operational cohort of treatment naive HIV infected women set up to transition patients onto ARV between 2003-2010. **Although guidelines for annual screening have been established, implementation has been poor - authors do not think over-diagnosis bias exists.** Among 2325 women screened at baseline, 1193 women had at least one smear >5.5 months after baseline smear. Women who had only one smear were more likely to have baseline intraepithelial lesion, more likely to be lost-to-follow-up or to have died than women with ≥2 smears (but no different by ART status or CD4+ count)**. Possible bias attributed to LTFU of women who were sicker during follow-up.**	Cox proportional hazards model. **Low risk**	Age, baseline CD4+, baseline smear result, smoking, baseline weight, time-varying ART. **Low risk**	Cytology on all; performed at National Health Laboratory Service - accredited by South Africa National Accreditation System. Women referred to colposcopy if cytology abnormal. Smear readers not blinded to previous smears which could have resulted in spuriously higher rates of premalignant lesions than if readers were blinded. Each smear was verified by second reader and all previous smears with higher/lower grade diagnosis to the current were then re-reviewed by a senior technologist. For women diagnosed with ASCUS (n=16), their subsequent smear was used instead of their baseline smear in longitudinal analysis of progression and regression. ASCUS diagnosed at endline were excluded. Medium risk of over diagnosis.	(Progression): Baseline CD4 <200 vs. >500	aHR=1.96 (95%CI: 1.33-2.88), adjusted for age, baseline smear result, smoking history and time-varying ART
Zeier, 2012	Retrospective cohort analysis of records from Colposcopy Clinic and Infectious Disease Clinic in Tygerberg Hospital, Cape Town, information collected from 2004-2009. Authors identified 1,960 cases that had LSIL at first abnormal smear, of these 1,720 had follow-up data available (12% LTFU); only women with FU visit > 6mths after first LSIL detected were included, but they were included if they experienced progression event <6mths. **Low risk**	Multivariable Cox proportional hazards regression model. **Low risk**	Age, CD4+ count at first LSIL, virological failure (2 consecutive HIV VL measurements of >1000 copies/ml at least 4 weeks apart) and excision treatment. ART considered as started before LSIL development (so no requirement for adjustment for duration). **Low risk.**	Cytology on all; quality of cytology assessed by determining the percentage of smears with endocervical cells present. Cytology result read by pathologist and checked by a second pathologist. **Low risk.**	n/a	n/a
Asia						
Chen, 2014	Population-based analysis conducted using the National Health Insurance Research Database, which covers approximately 99% of people living in Taiwan. Of ~17,000 patients with HIV in the database, ~10% are women (possible underrepresentation of women attending services). Data on HIV factors from pharmacy refill record.	Multivariable Cox proportional hazards regression model. **Low risk**	Age at HIV diagnosis, income, urbanization level, occupation, drug dependence, treated OIs, history of STI, frequency of pap test after HIV diagnosis. No information on nadir or current CD4+. **Medium risk.**	CIS/ICC determined if diagnosis reached at least twice in the medical records from outpatient or hospital claims during study period. No histopathological diagnosis. Low-medium risk.	Long duration ART (>3yrs) vs. ART-naïve/ART≤6 months	aHR=0.03 (95%CI:0.01-1.16)

First author, year	Participant selection, ART status and LTFU	Statistical methods used	Adjustment for confounding	Endpoint assessment	Other significant findings related to HIV	
					**(Outcome) Comparison groups**	**Effect estimate, adjustment**
Latin America						
Kreitchmann, 2013	Enrolled all WLHIV referred for gynaecological exam at reference center for STI/AIDS in Porto Algere, Brazil. Of 898 women with a baseline pap results, 388 (43%) returned for follow-up pap smear. **Possible exclusion bias**.	Multivariable Cox proportional hazards regression model. **Low risk**	Age, CD4+ count, log viral load, years at school. Not adjusted for sexual behaviour. **Medium risk.**	Cytology on all; all pap smears evaluated in one reference laboratory and interpreted by a certified cytopathologist. Approx. 10% of negative pap smears, chosen at random, and all positive smears for any cytological abnormality were independently interpreted by another cytopathologist. Low risk	(Incidence): Current CD4 ≤200 vs. >500	aHR=3.0 (95%CI:1.2-7.2), adjusted for Age, ART, viral load, race, education
					(Incidence): Log viral load (copies/ml)	aHR=1.4 (1.0-1.9), adjusted for Age, ART, CD4+, race, education
Europe/North America (in alphabetical order)						
Blitz, 2013	Women recruited from 28 community based or tertiary care centres across Canada; 58% Canadian, 31% from HIV-endemic country, from 1993-2002. There was 29% loss to follow-up after baseline visit. **Medium risk of selection bias. 19% were on HAART at start of study; 64% by end of study -includes initiators**	Multistate time-homogenous Markov models used to model bidirectional transitions through different disease states by individuals over time; assumes that transition from one state to another is constant over time and the probability of transition depends on time between observations, rather than the overall time of observation. **Low risk**	Unadjusted -univariate results presented only. Increased risk of progression and increased likelihood of regression among women with >5 LTSP. **High risk**.	Cytology on all. Women with abnormal findings referred for colposcopy and biopsy and treatment as necessary. Only cytology results used as endpoint. **Medium risk of reporting bias. Sensitivity analysis which groups ASCUS with normal found similar results.**	n/a	n/a
Clifford, 2016	Swiss HIV cohort study (SHCS): nationwide prospective cohort enrolling PLHIV ≥16yrs. Case control study including cases of CIN2/3 and ICC record in the SHCS and matched to ≤CIN1 controls using incidence density sampling, matched on age, location, HIV transmission category and year of enrolment. CIN2/3 and ICC cases routinely recorded in the SHCS but additional cases identified through record linkage with 8 cantonal cancer registries. **Low risk**	Logistic regression. Not included in meta-analysis	Cases and controls matched on HIV-transmission category (IDU, heterosexual/other), age and calendar year at enrolment and place of recruitment. Adjusted for Nadir CD4+. **Low risk**	Cases of CIN2/3 and of ICC are routinely recorded in the SHCS. Additional cases were identified through record linkage with eight cantonal cancer registries. Unclear if there was histopathological diagnosis.	(CIN2/3 Incidence): per 100 cell/µl decrease in nadir CD4+	OR=1.15, 95%CI: 1.08-1.22
					(CIN2/3 Incidence): per 100 cell/µl decrease in current CD4+ (at time of CIN2/3 diagnosis)	OR=1.10, 95%CI: 1.04-1.16
					(ICC Incidence): per 100 cell/µl decrease in nadir CD4+	OR=1.19, 95%CI: 0.88-1.60
					(ICC Incidence): per 100 cell/µl decrease in current CD4+ (at time of CIN2/3 diagnosis)	OR=1.16, 95%CI: 0.89-1.51

First author, year	Participant selection, ART status and LTFU	Statistical methods used	Adjustment for confounding	Endpoint assessment	Other significant findings related to HIV	
					**(Outcome) Comparison groups**	**Effect estimate, adjustment**
Del Mistro, 2004	All HIV infected women attending the Infectious Disease Units of Vicenza and Padova City Hospitals were offered gynaecologic consultation from 1994-2002. HIV infection acquired by injection drug use or heterosexual contact (in near equal proportions). 201 women with ≥1 follow-up visit were included, but no information on numbers at baseline. Mortality during FU was 9.5%, cause of death linked to HIV infection, except for 2 which were related by hysterectomy complications and breast cancer. **Unclear Risk?**	Unadjusted analysis.	Unadjusted analysis undertaken by current authors. **High risk.**	Cytology on all; colposcopy driven biopsy for high grade SILs. Unclear if quality checks used for cytology reading. **Unclear risk.**	-	-
Ellerbrock, 2000	Prospective cohort study of women enrolled in the New York Cervical Disease Study, recruited from HIV clinics between 1991-1996. Women recruited from HIV clinics directly by health care provider, without regard of risk factors for cervical disease or clinical HIV status. Inclusion of women with no evidence of SIL at baseline –all women had follow-up pap smear. Enrolled women likely to report history of prostitution (24%) and intravenous drug use (44%). Study conducted prior to introduction of protease inhibitors. **High risk – possible bias given the population not generalizable and period effect (early ARV use).**	Cox proportional hazard model. Assumption of proportionality was tested and met in multivariate analysis. **Low risk.**	Age, smoking, transient and persistent HPV, CD4 at enrolment. **ART was included as a time-dependent variable. Low risk**	Cytology on all, confirmed by histology (428 biopsies read). **Low risk of misclassification.**	n/a	n/a
Guiguet, 2009	Large prospective hospital cohort - French Hospital Database on HIV (FHDH-ANRS CO4). Patients infected with HIV-1 were eligible if they had never received ART before enrolment in the FHDH cohort, had never been included in a double-blind clinical trial, and had not been diagnosed with cancer before 1998. Patients were followed up until diagnosis of cancer, death, the end of follow-up, or Dec 31, 2006, whichever occurred first. **Low risk**	Poisson regression. Low risk.	Age, sex and HIV transmission group, and sub-Saharan origin. ART was entered on an intention to-treat approach, ignoring changes to the initial regimen. All cumulative durations were calculated over the entire follow-up in the FHDH cohort; time-varying covariables were updated at the beginning of every month. Limited data on alcohol or smoking.	Clinical events were recorded with International Classification of Diseases (ICD) definitions (version 10). Disease misclassification could have occurred; however, because of the magnitude of person-time in the cohort, the probability of being wrongly classified as having a malignancy will be negligible, limiting bias for the rate ratios. No histopathological diagnosis. Low-medium risk.	(ICC Incidence) Current CD4 (per $\log_2$ increase)	RR=0.70 (0.60-0.80)

First author, year	Participant selection, ART status and LTFU	Statistical methods used	Adjustment for confounding	Endpoint assessment	Other significant findings related to HIV	
					**(Outcome) Comparison groups**	**Effect estimate, adjustment**
Heard, 2002	Prospective study among HIV positive women attending outpatient HIV clinics in Paris initiated in 1993, during period 1993-1999. All enrolled women included in follow-up visit. **Period effect - unclear risk.**	Multivariate analysis of lesion regression and risk factors performed using Cox's proportional hazard model. **Low risk**	HAART was considered as time-dependent variable using intention-to-continue treatment approach. Women starting HAART during follow-up were classified as off-HAART until time of first prescription and on-HAART thereafter. Other adjustments for CD4+ count at detection, calendar period of CIN detection (1997-1999 vs. 1996-1996), and lesion grade. **Low risk**	Cytology on all, standardised colposcopic exam with biopsy if necessary. Smears and biopsies read by same pathologist. CIN defined as high grade on basis of histologically confirmed high grade CIN or low grade CIN associated with smear showing HGSIL, similar for low grade. Normal smear and colposcopy, minor colposcopic changes associated with normal smear were considered as no evidence of CIN. Biopsies taken for 57% of all participants. **Possible reader bias?**	n/a	n/a
Heard, 2006	Prospective study enrolling women attending gynaecology outpatient clinic of the HIV dept. of Hôpital Européen Georges Pompidou and Hôpital Cohin, Paris between 1993-2005. Women with SIL diagnosed before or at time of enrolment were excluded, including those with abnormal colposcopic finding at enrolment. All enrolled women included in follow-up visit. **Low risk**	Multiple Cox regression model accounting for duration of follow-up. Relative risk reported. **Low risk**	Age, ethnicity, smoking, LTSP, contraception, condom use, CD4+ count, inclusion period. Hormonal contraception, condom use, CD4+ and HAART as time-dependent variables. **Low risk**	Cytology on all; patients with major colposcopic abnormalities or high grade SIL underwent a biopsy, unless they refused or were not compliant for follow-up. All smears and biopsies read by the same pathologist. **Possible reader bias?**	n/a	n/a
Kim, 2013	Twenty-year Retrospective study among HIV infected women cared for at Strong Memorial Hospital AIDS Centre with ≥2 pap smears between 1991-2011; 800-1061 individuals with HIV were followed of which 30% were women. Mean nadir CD4=206 cells per µl. Of the available data among 313 WLHIV in the database, 68 (22%) women were excluded: 38 had a history of hysterectomy; 1 had no data on ART; 29 had < 2 follow-up pap smears (17 of these were considered lost to follow-up; 12 had only recently entered the cohort). **Low risk.**	Cox proportional hazards model allowing for the possibility of a patient having multiple events. Sandwich estimator used to adjust for the correlation. **Low risk**	Time-dependent covariates assessed at visit prior to event or censoring:CD4+ count, duration of HIV infection, menopausal status, drug use, smoking LTSP and condom use not associated in univariate analysis **Low risk**	Cytology on all (Bethesda 1988 and 2001). Women with abnormal pap tests were referred to gynaecology clinic for colposcopy and further management. Unclear whether only cytology results used. **Medium risk.**	(Progression): Per 100 CD4 cells/mm^3 increase	aHR=0.91 (95%CI: 0.86-0.96), adjusted for ART, Duration of HIV infection, menopausal status, drug use, smoking
Lehtovirta, 2006	Retrospective study of all HIV positive women attending Dept. of Obstetrics and Gynaecology in the University of Helsinki from 1989 to 2003, including all WLHIV followed up with >1 visit; 65% of Finnish, 21% of African origin, origin. Women with only 1 visit were excluded from analysis 9(n=29; 21%) and 3 women with hysterectomy. **Low-medium risk.**	Cox proportional hazard analysis used for calculating Relative Risk. **Unclear risk.**	Age. No adjustment for ART duration during follow-up or sexual behaviour. **High risk.**	Cytology on all; in event of LSIL+, patients had colposcopy and biopsy	n/a	n/a

First author, year	Participant selection, ART status and LTFU	Statistical methods used	Adjustment for confounding	Endpoint assessment	Other significant findings related to HIV	
					**(Outcome) Comparison groups**	**Effect estimate, adjustment**
Lillo, 2001	HIV positive women recruited from a patient care program that included gynaecologic monitoring from 1995-1997. All enrolled women included in follow-up visit. Low risk.	Odds Ratios estimated using logistic regression. **High risk- does not take into account changes over time.**	CD4+ count, HIV RNA and gynaecologic treatment. No adjustment for duration on ART or sex behaviour. **High risk**	Cytology on all; slides with abnormal smears were regularly seen by 2 cytopathologists and 2 physicians. 53 women (33%) had colposcopy driven biopsy at baseline: 68% had LSIL and 13% had HSIL. **Low risk or reporting bias.**	n/a	n/a
Massad, 2004	Prospective follow-up of ART initiators in the Women's Interagency HIV Study (WIHS) from 1994-2002.	Cox proportional hazards model.	Age at diagnosis of CIN1, parity, current tobacco smoking, number of sexual partners (both lifetime and within the preceding 6 months), HPV risk type at diagnosis of CIN1, CD4 count, HIV RNA level, cytology result at diagnosis of CIN1, colposcopic examination adequacy, time-varying ART	All CIN 1 was based on histology. Slides were not centrally reviewed, but interrater correlation of grading across sites was moderate to strong. Regression from CIN1 was defined either as normal colposcopy and at least 1 smear read as negative for epithelial abnormality or as 3 consecutive negative smears if repeat colposcopy was not performed. **Low risk**	(Regression) CD4 <200 vs >500	HR=0.49, 95%CI: 0.24-1.01
Minkoff, 2001	HIV positive women enrolled in six clinical consortia: Bronx, Brooklyn, Chicago, Los Aneles, San Francisco, Washington between 1994-1995. Among 2,059 women enrolled, 1,779 (86%) had at least one study visit after Oct 1995 when HAART become available. This study was limited to women with detectable high-risk HPV type (n=741). **Selection bias of women with HR-HPV positive lesions**.	Odds Ratios estimated using logistic regression. As each participant could contribute >1 pair of smears, inferences were based on robust statistical methods that adjust for correlation inherent in such repeated measures. **High risk**- does not take into account changes over time.	CD4+ count and pap smear status at baseline. No adjustment for ART duration or sex behaviour. **High risk**	Cytology (Bethesda 1994). All smears read by two cytotechnologists who were blinded to participants HIV status. All abnormal smears and 10% of all negative smears were confirmed by cytopathologist. **Low risk**	n/a	n/a
Minkoff, 2010	Prospective follow-up of ART initiators in the Women's Interagency HIV Study (WIHS) from 1994-2002; analysis restricted to women initiating ART with ≥2 semi-annual visits during the 2.5 years just prior to HAART initiation and ≥2 semi-annual visits during the 2.5 years immediately following HAART initiation. **Selection bias of ART initiators.**	Within-woman analysis using random effects model -controlled for the fact that the data involved repeat observations of the same women over time and each woman acted as their own comparison group (after compared to before ART initiation). **Low risk**	Adjusted for time-dependent variable, CD4+ count pre- and post-HAART. Adjustment for other cofactors had no impact on findings (age, number sex partners in last 6 months, smoking, race/ethnicity) **Low risk**	Cytology on all. Oncogenic SIL (SIL that was HR-HPV DNA positive) was used as endpoint. HPV DNA testing was performed using cervicovaginal lavage sample tested on PCR. There is a possibility that some SIL lesions could have been missed (incorrectly classified as HR-HPV negative). **However, when analysis was repeated with any SIL, results were similar. Low risk.**	(Regression): Adherence vs. non-adherence	aHR=3.75 (95%CI: 1.43-9.88), adjusted for treatment of CIN using a time-dependent variable, and CD4+ count at the start of each period
Paramsothy, 2009	HIV Epidemiology Research Study (HERS): a prospective cohort study of women with HIV without AIDS–defining conditions were selected between 1996-2000; study sites in Bronx, Providence and Detroit and Baltimore. There were 871 women with HIV enrolled in HER study. The study is restricted to 537 (62%) women who were diagnosed with HIV before enrolment in the HER study and had at least 2 study visits (~38% LTFU. **Low-medium risk (LTFU >20%).**	Cox proportional hazards model. Women never on HAART had time calculated from first until their last study visit. Women on HAART; first study visit when they report HAART use until their last study visit. Women on HAART were placed in the model for the time before they started HAART as pre-HAART; measured from first study visit until visit before HAART use	CD4+ count, HPV DNA status, baseline pap test result. Intent-to-treat analysis: once a woman reported HAART use, she was considered to be on HAART through her last visit; effect of discontinuation, changes in regimen or adherence were not evaluated. Adjusted for	Cytology on all (Bethesda 1988). Women with abnormal pap in Bronx and Providence were referred for colposcopy. In Detroit and Baltimore, all women received colposcopy. No information about quality measures for reading. **Unclear risk.**	n/a	n/a

First author, year	Participant selection, ART status and LTFU	Statistical methods used	Adjustment for confounding	Endpoint assessment	Other significant findings related to HIV: (Outcome) Comparison groups	Other significant findings related to HIV: Effect estimate, adjustment
		(pre-HAART combined with never HAART as comparison group). **Low risk**	time-varying CD4+. **Medium risk**			
Sirera, 2008	Retrospective cohort study including all patients in the database of the HIV Clinical Unit of the Germans Trias i Pujol University Hospital between 1997-2006, including only women with CD4+ >350 cells/mm3 at baseline. **High selection bias.**	Multivariate proportional hazard regression (Cox regression) using log-rank test. **Low risk**	CD4+ count, number of partners. Not adjusted for time-varying ART. Medium risk	Cytology on all. Most samples were checked by two cytopathologists. When a patient was diagnosed with LSIL+, a colposcopy and biopsy were proposed to verify cytology result. **Low risk.**	n/a	n/a
Soncini, 2007	Screening data from pap smears and colposcopic exams were collected from HIV positive women at Colposcopy and Cervical Pathology Service at Parma University Hospital between 1993-2003 within a year of being admitted to a screening and early-diagnosis program for cervical cancer. All patients without prior diagnosis of CIN at first visit were enrolled. 81% of Italian origin, 14% of African origin. **Low risk**	Cox regression model and log-rank test. **Low risk**	HAART was considered as time-dependent variable adjusted for CD4+ at first visit. Not adjusted for sexual behaviour. **Low-medium risk**	Cytology and colposcopy (93% of visits had combined cytology and colposcopy, remainder were colposcopy only). If pap and/or colposcopy reveal abnormality, targeted biopsy performed. Cytology and histology reviewed by gynaecologic pathologists at the hospital. Colposcopy by two expert colposcopists, according to IFCPC classification. **Low risk.**	(Incidence): Low vs. high CD4 at enrolment (unclear reporting)	aHR=2.38 (95%CI:1.44-3.96), adjusted for time-dependent ART
Schuman, 2003	HERS study: women 16-55yrs eligible if they reported injection use or high risk sex and no history of AIDS defining illness between 1993-1995. 774 (89%) of 871 women enrolled in HERS study had follow-up data and were included in the study. **Possible selection bias for a high risk population.**	Discrete time survival analysis with complementary log-log model link allowing a Relative Risk interpretation of the coefficients. Repeated measures multivariate logistic regression models used for progression/regression, accounting for within-subject correlation. **Low risk**	Cofactors permitted to have different values at each visit. Adjusted for time (visit), study site, age, race, education. No association with sexual activity for incidence (no adjustment for progression or regression). Not possible to model ART duration ART; reported ART at each visit was considered; would not have taken into account new, intermittent or prolonged duration on ART. Medium-**High risk**	Cytology (Bethesda 1988) on all. A senior cytopathologist read all tests originally classified as abnormal and 10% of the normal. **Low risk.**	(Incidence): CD4 <200 vs >500 at baseline	aRR=2.13 (95%CI: 1.27-3.64), adjusted for time (visit), site and age
					(Progression): CD4 <200 vs >500 at baseline	aRR=1.76 (95%CI: 1.09-2.84), adjusted for time (visit), site and age, race and education
					(Regression): HIV-1 viral load (status at previous visit)	aRR=0.78 (95%CI: 0.65-0.94), adjusted for time (visit), site and age, race and education

NR=not reported; LTFU=loss to follow-up; ARV=antiretroviral; BMI=body mass index; STI=sexually transmitted infection; NHLS=National Health Laboratory Service; aHR=adjusted Hazard Ratio; LTSP=lifetime sexual partners; VIA/VILI=visual inspection using acetic acid or Lugol's iodine; HC-II=Hybrid Capture II; AFS=age at first sexual intercourse; LTSP=lifetime sexual partners; ASCUS= atypical squamous cells of undetermined significance; LSIL=low-grade squamous intraepithelial lesions; ; HSIL=high-grade squamous intraepithelial lesions; VL=viral load; OI=opportunistic infection; CIS=carcinoma *in situ*; ICC=invasive cervical cancer; PLHIV=people living with HIV; IDU=injecting drug user; FU=follow-up; HAART=highly active antiretroviral therapy; LTSP=lifetime sexual partners.

Supplementary Figure 4. Meta-analysis of HR-HPV and HSIL-CIN2+ prevalence among prolonged duration ART users (≥2 years duration) compared to ART-naïve or short duration ART users (<2 years)

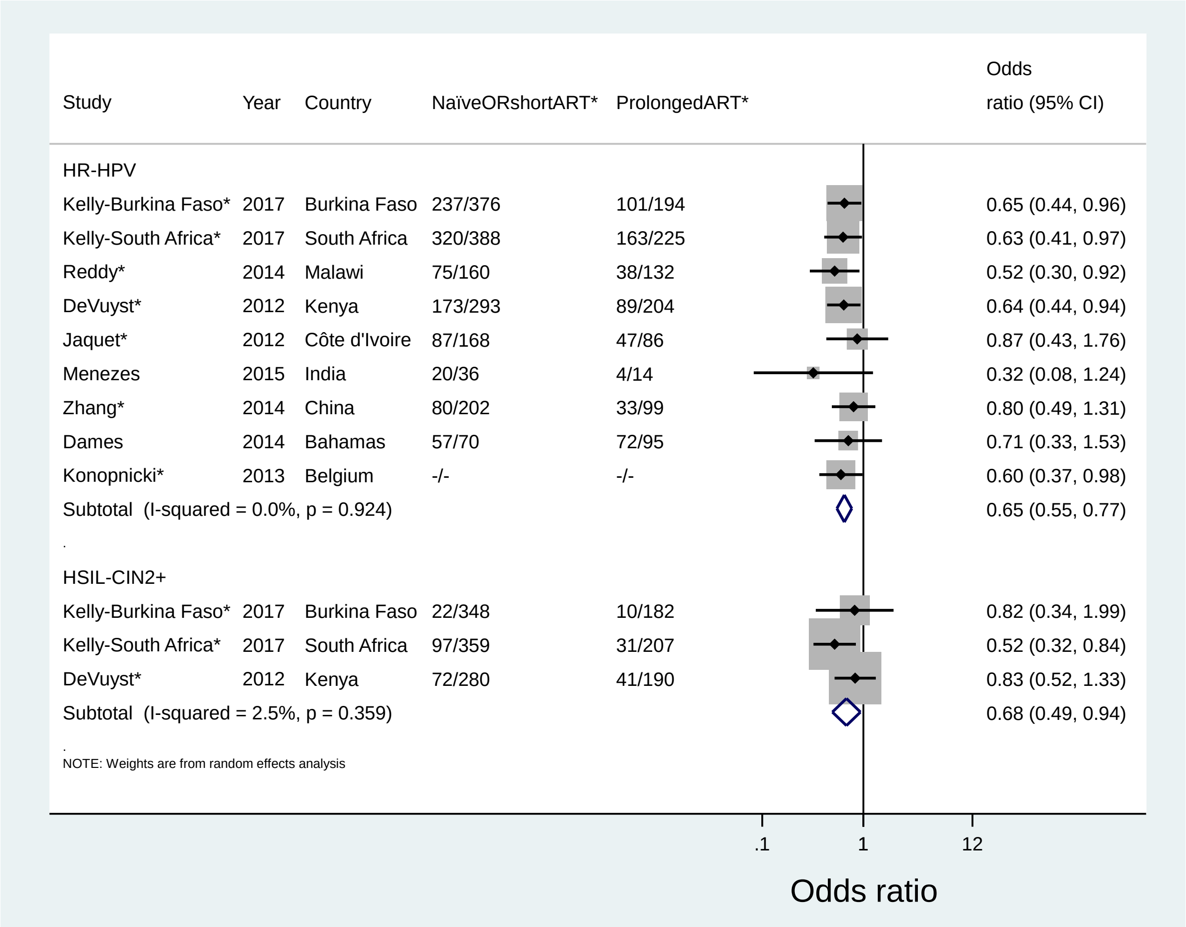

*HR-HPV or HSIL-CIN2+ prevalence among prolonged ART users (≥2 years) compared to a combined group of ART-naive or short-duration users (<2 years)

Supplementary Table 7. HR-HPV and HSIL-CIN2+ prevalence according to ART status

	**ART-naïve n/N (%)**	**ART <2 years n/N (%)**	**ART ≥2 years n/N (%)**
**HR-HPV prevalence**			
Kelly-Burkina Faso (2017)	95/158 (60.1)	142/218 (65.1)	101/194 (52.1)
Kelly-South Africa (2017)	173/209 (82.8)	147/179 (82.1)	163/225 (72.4)
Reddy (2014)	18/45 (40.0)	57/115 (49.6)	38/132 (28.8)
DeVuyst (2012)	71/122 (58.2)	102/171 (59.7)	89/204 (43.6)
Jaquet (2012)	33/64 (51.6)	54/104 (51.9)	47/86 (54.7)
Menezes (2015)	14/26 (53.8)	6/10 (60.0)	4/14 (28.6)
Zhang (2014)	37/108 (34.3)	43/94 (45.7)	33/99 (33.3)
Dames (2014)	24/31 (77.4)	33/39 (84.6)	72/95 (75.8)
Konopnicki (2013)	NR	NR	NR
**HSIL-CIN2+ prevalence**			
Kelly-Burkina Faso (2017)	6/142 (4.2)	16/206 (7.8)	10/182 (5.5)
Kelly-South Africa (2017)	50/198 (25.3)	47/161 (29.2)	31/207 (15.0)
DeVuyst (2012)	27/120 (22.5)	45/160 (28.1)	41/190 (21.6)

Supplementary Figure 4. Meta-analysis of cervical lesion (any cytological/histological grade) prevalence among ART users compared to ART-naïve among 26 studies

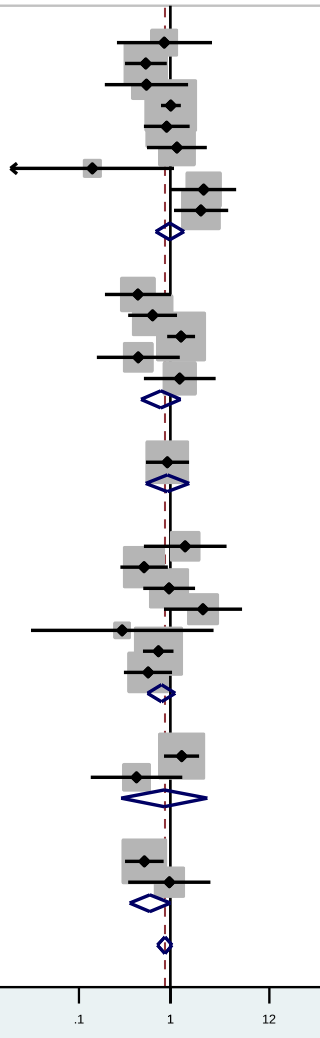

Study	Year	Country	ART	ARTnaive	Odds ratio (95% CI)
CIN2+					
Kelly-BF*	2017	Burkina Faso	26/388	6/142	0.86 (0.26, 2.83)
Kelly-SA*	2017	South Africa	78/368	50/198	0.54 (0.32, 0.91)
Memiah	2015	Kenya	4/108	38/578	0.55 (0.19, 1.56)
Huchko	2014	Kenya	142/1604	145/1581	1.01 (0.79, 1.30)
De Vuyst*	2012	Kenya	86/350	27/120	0.91 (0.51, 1.62)
Mabeya	2012	Kenya	32/100	14/49	1.18 (0.56, 2.49)
Feng*	2012	China	13/193	15/108	0.14 (0.02, 1.09)
De Andrade	2011	Brazil	10/90	12/250	2.31 (1.02, 5.23)
Sahasrabuddhe	2010	India	17/70	26/201	2.16 (1.09, 4.28)
Subtotal (I-squared = 58.7%, p = 0.013)					0.99 (0.69, 1.41)
.					
HSIL+					
Ezechi	2014	Nigeria	15/374	10/116	0.44 (0.19, 1.01)
Patrelli	2013	Italy	40/127	28/67	0.64 (0.35, 1.18)
Firnhaber	2010	South Africa	127/656	55/354	1.31 (0.92, 1.86)
Mogtomo	2009	Cameroon	8/35	14/35	0.44 (0.16, 1.26)
Kitchener	2007	Europe**	NR	NR	1.26 (0.51, 3.11)
Subtotal (I-squared = 62.0%, p = 0.033)					0.79 (0.48, 1.30)
.					
LSIL+					
Mangclaviraj	2008	Thailand	36/226	27/159	0.93 (0.54, 1.60)
Subtotal (I-squared = .%, p = .)					0.93 (0.54, 1.60)
.					
ASCUS+					
Chakravarty	2016	India	43/162	5/25	1.45 (0.51, 4.09)
Katumba	2016	South Africa	180/330	42/60	0.51 (0.28, 0.93)
Liu	2015	Tanzania	108/1242	11/123	0.97 (0.51, 1.86)
Prabha Devi	2013	India	11/107	7/145	2.26 (0.85, 6.04)
Marchetti	2012	Italy			0.30 (0.03, 2.95)
Chalermchockcharoenkit	2011	Thailand	53/395	74/426	0.74 (0.50, 1.08)
Drougel-Vey	2007	France	41/105	179/315	0.57 (0.31, 1.04)
Subtotal (I-squared = 39.5%, p = 0.128)					0.80 (0.56, 1.13)
.					
CIN1+					
Jaquet	2014	Côte d'Ivoire	106/2267	26/731	1.33 (0.86, 2.06)
Oliveira	2010	Brazil	7/38	9/26	0.43 (0.13, 1.35)
Subtotal (I-squared = 69.5%, p = 0.070)					0.86 (0.29, 2.54)
.					
Visual inspection/colposcopy					
Gedefaw	2013	Ethiopia	224/304	121/144	0.52 (0.32, 0.84)
Curry	2012	USA	12/49	8/32	0.97 (0.35, 2.73)
Subtotal (I-squared = 13.9%, p = 0.281)					0.60 (0.36, 1.00)
.					
Overall (I-squared = 52.5%, p = 0.001)					0.87 (0.72, 1.05)

NOTE: Weights are from random effects analysis

Supplementary Table 7. Summary of studies of the association of ART with cervical lesion (any grade) prevalence

First author, year	Study design	Location	Enrolment period	Sample size	% ART users	CD4+ count, cells per µl Median [IQR] or mean (SD or range)	Lesion definition	Lesion prevalence	Comparison group	Crude Odds Ratio (95% CI)	Adjusted Odds Ratio (95% CI)
≥ASCUS											
Chakravarty, 2016[55]	Cross-sectional	India-Varanasi	Dec 2010-June 2012	187	86.6%	368 [239-524]	≥ASCUS	25.7%	ART vs. ART-naïve	1.45 (0.51-4.09)	-
Katumba, 2016[56]											
Liu, 2015[57]	Cross-sectional	Tanzania-Dar-es-Salaam	Dec 2006-Aug 2009	1365	91.0%	164 [80-257]	≥ASCUS	8.7%	ART vs. ART-naïve	0.97 (0.51-1.86)	-
Prabha Devi, 2013[58]											
Marchetti, 2012[59]	Cross-sectional	Italy-Milan	Jan 2009-Feb 2011	97	NR	205 [120-288]	≥ASCUS	37%	HAART vs. HAART-naïve	-	0.30 (0.03-2.94)[a]
Chalermchockcharoenkit, 2011[60]	Cohort	Thailand-Salaya	Jan 2004-Dec 2009	821	48.1%	324 [range:2-999]	≥ASCUS	15.5%	HAART vs. HAART-naïve	0.74 (0.50-1.08)	-
Drougel-Vey, 2007[61]	Case-control	France-Marseille	1991-2004	420	25.0%	341 [180-494]	≥ASCUS	52.4%	ART vs. ART-naïve	-	0.57 (0.31-1.04)[b]
≥LSIL											
Mangclaviraj, 2008[62]	Cross-sectional	Thailand-Bangkok	Jan 2002-Dec 2005	385	58.7%	~50% of women had CD4+ between 200-499 cells per µl at time of pap smear	≥LSIL	11.2%	ART vs. ART-naïve	0.93 (0.54-1.60)	-
≥CIN1											
Jaquet, 2014[63]	Cross-sectional	Côte d'Ivoire-Abidjan	Aug 2009-Nov 2010	2998	75.6%	ART users: 439 [282-616]; ART-naïve: 491 [361-640]	≥CIN1+	4.4%	ART vs. ART-naïve	1.33 (0.86-2.06)	-
Oliveira, 2010[64]	Cross-sectional	Brazil-Bahia	May 2006-May 2007	64	59.4%	Mean: 644 [SD ±551]	≥CIN1+	25.0%	ART vs. ART-naïve	0.43 (0.13-1.35)	-
Abnormality on Visual Inspection/Colposcopy											
Gedefaw, 2013[65]	Cross-sectional	Ethiopia-Hawassa, Yirga Alem, Sodo,	Oct 2012-Feb 2013	448	67.9%	Mean: 172 [SD ±86]	Visual Inspection/ colposcopy abnormal	22.1%	HAART vs. HAART-naïve	-	0.52 (0.35-0.92)[c]
Curry, 2012[66]	Retrospective analysis	USA-Boston	2002-2008	81	60.5%	376	Visual Inspection/ colposcopy abnormal	24.7%	HAART vs. HAART-naïve		

[a]Adjusted for age, sexual activity, CDC staging**, Nadir CD4+**, HR-HPV infection; [b]adjusted for age, SIL in pre-HAART period, CD4+; [c]adjusted for age, education, employment, parity, history of pelvic infection, and STI , age at first sexual intercourse, age at first marriage, lifetime number of sex partners

Supplementary Table 8. Meta-analysis of the association of ART with cervical lesion (any cytological or histological grade) prevalence

Outcome	N studies	Pooled OR (95%CI)	I^2	P
Histology				
CIN2+	9	0.99 (0.69-1.41)	58.7%	0.013
CIN1+	2	0.86 (0.29-2.54)	69.5%	0.070
Cytology				
HSIL+	5	0.79 (0.48-1.30)	62.0%	0.033
LSIL+	1	0.93 (0.54-1.60)	-	-
≥ASCUS	7	0.80 (0.56-1.13)	39.5%	0.128
Visual Inspection/colposcopy	2	0.60 (0.36-1.00)	13.9%	0.281

Supplementary Figure 5. Funnel plot of publication bias among studies evaluating the association of ART with HR-HPV, cervical lesions and invasive cervical cancer *

A. HR-HPV Prevalence (OR=Odds Ratio)

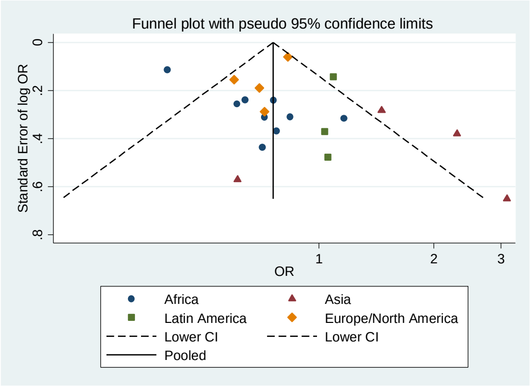

B. HSIL-CIN2+ prevalence (OR=Odds Ratio)

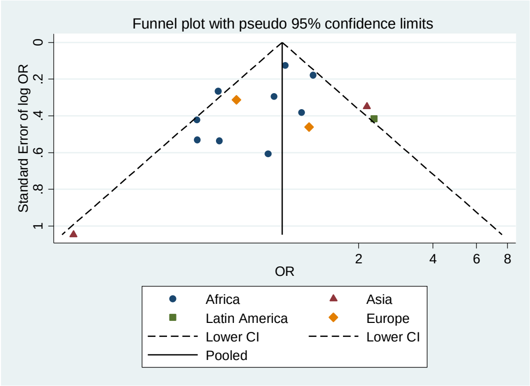

C. SIL-CIN incidence

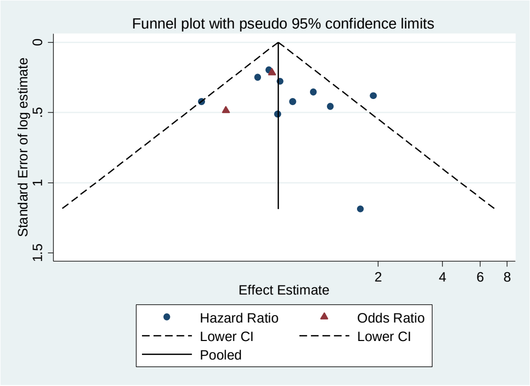

D. SIL progression

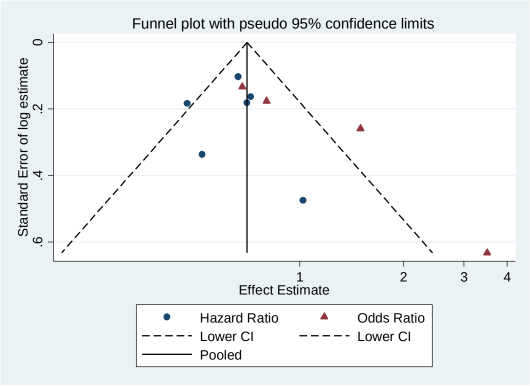

E. SIL-CIN regression

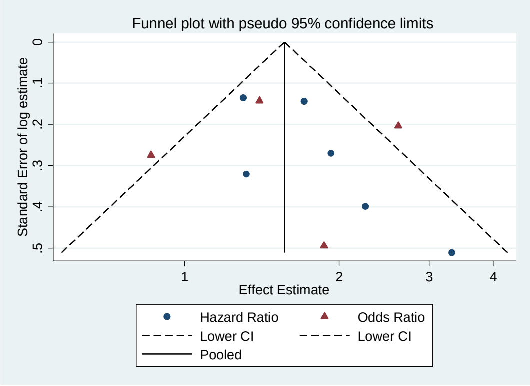

F. Invasive cervical cancer incidence

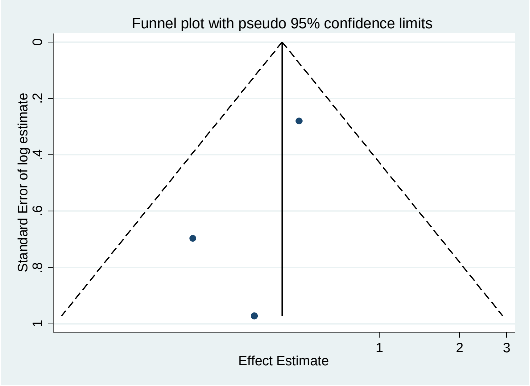

References

1. Kelly HA, Sawadogo B, Chikandiwa A, et al. Epidemiology of high-risk human papillomavirus and cervical lesions in African women living with HIV/AIDS: effect of anti-retroviral therapy. Aids. 2017;31(2):273-85.
2. Zeier MD, Botha MH, Engelbrecht S, et al. Combination antiretroviral therapy reduces the detection risk of cervical human papilloma virus infection in women living with HIV. Aids. 2015;29(1):59-66.
3. Mane A, Nirmalkar A, Risbud AR, Vermund SH, Mehendale SM, Sahasrabuddhe VV. HPV genotype distribution in cervical intraepithelial neoplasia among HIV-infected women in Pune, India. PLoS ONE [Electronic Resource]. 2012;7(6):e38731.
4. Ezechi OC, Ostergren PO, Nwaokorie FO, Ujah IA, Odberg Pettersson K. The burden, distribution and risk factors for cervical oncogenic human papilloma virus infection in HIV positive Nigerian women. Virology Journal. 2014;11:5.
5. Reddy D, Njala J, Stocker P, et al. High-risk human papillomavirus in HIV-infected women undergoing cervical cancer screening in Lilongwe, Malawi: a pilot study. Int J STD AIDS. 2015;26(6):379-87.
6. Rositch AF, Gravitt PE, Tobian AA, et al. Frequent detection of HPV before and after initiation of antiretroviral therapy among HIV/HSV-2 co-infected women in Uganda. PLoS One. 2013;8(1):e55383.
7. De Vuyst H, Mugo NR, Chung MH, et al. Prevalence and determinants of human papillomavirus infection and cervical lesions in HIV-positive women in Kenya. British Journal of Cancer. 2012;107(9):1624-30.
8. Jaquet A, Horo A, Charbonneau V, et al. Cervical human papillomavirus and HIV infection in women of child-bearing age in Abidjan, Cote d'Ivoire, 2010. Br J Cancer. 2012;107(3):556-63.
9. Veldhuijzen NJ, Braunstein SL, Vyankandondera J, et al. The epidemiology of human papillomavirus infection in HIV-positive and HIV-negative high-risk women in Kigali, Rwanda. BMC Infect Dis. 2011;11:333.
10. Menezes LJ, Poongulali S, Tommasino M, et al. Prevalence and concordance of human papillomavirus infection at multiple anatomic sites among HIV-infected women from Chennai, India. Int J STD AIDS. 2016;27(7):543-53.
11. Zhang HY, Fei MD, Jiang Y, et al. The diversity of human papillomavirus infection among human immunodeficiency virus-infected women in Yunnan, China. Virol J. 2014;11:202.
12. Aggarwal R, Sachdeva RK, Naru J, Suri V, Sharma A, Nijhawan R. HPV genotyping in north Indian women infected with HIV. Int J Gynecol Pathol. 2012;31(5):475-81.
13. Rocha-Brischiliari SC, Gimenes F, de Abreu AL, et al. Risk factors for cervical HPV infection and genotypes distribution in HIV-infected South Brazilian women. Infect Agent Cancer. 2014;9(1):6.
14. Dames DN, Blackman E, Butler R, et al. High-risk cervical human papillomavirus infections among human immunodeficiency virus-positive women in the Bahamas. PLoS One. 2014;9(1):e85429.
15. Grinsztejn B, Veloso VG, Levi JE, et al. Factors associated with increased prevalence of human papillomavirus infection in a cohort of HIV-infected Brazilian women. Int J Infect Dis. 2009;13(1):72-80.
16. Konopnicki D, Manigart Y, Gilles C, et al. Sustained viral suppression and higher CD4+ T-cell count reduces the risk of persistent cervical high-risk human papillomavirus infection in HIV-positive women. Journal of Infectious Diseases. 2013;207(11):1723-9.
17. Blitz S, Baxter J, Raboud J, et al. Evaluation of HIV and highly active antiretroviral therapy on the natural history of human papillomavirus infection and cervical cytopathologic findings in HIV-positive and high-risk HIV-negative women. Journal of Infectious Diseases. 2013;208(3):454-62.

18. Minkoff H, Zhong Y, Burk RD, et al. Influence of adherent and effective antiretroviral therapy use on human papillomavirus infection and squamous intraepithelial lesions in human immunodeficiency virus-positive women. Journal of Infectious Diseases. 2010;201(5):681-90.
19. Fife KH, Wu JW, Squires KE, Watts DH, Andersen JW, Brown DR. Prevalence and persistence of cervical human papillomavirus infection in HIV-positive women initiating highly active antiretroviral therapy. J Acquir Immune Defic Syndr. 2009;51(3):274-82.
20. Memiah P, Makokha V, Mbuthia W, et al. Epidemiology of Cervical Squamous Intraepithelial Lesions in HIV Infected Women in Kenya: a cross-Sectional Study. Afr J Reprod Health. 2015;19(1):133-9.
21. Huchko MJ, Leslie H, Sneden J, et al. Risk factors for cervical precancer detection among previously unscreened HIV-infected women in Western Kenya. International Journal of Cancer. 2014;134(3):740-5.
22. Mabeya H, Khozaim K, Liu T, et al. Comparison of conventional cervical cytology versus visual inspection with acetic acid among human immunodeficiency virus-infected women in Western Kenya. J Low Genit Tract Dis. 2012;16(2):92-7.
23. Ezechi OC, Pettersson KO, Okolo CA, Ujah IA, Ostergren PO. The association between HIV infection, antiretroviral therapy and cervical squamous intraepithelial lesions in South Western Nigerian women. PLoS One. 2014;9(5):e97150.
24. Firnhaber C, Van Le H, Pettifor A, et al. Association between cervical dysplasia and human papillomavirus in HIV seropositive women from Johannesburg South Africa. Cancer Causes & Control. 2010;21(3):433-43.
25. Mogtomo ML, Malieugoue LC, Djiepgang C, Wankam M, Moune A, Ngane AN. Incidence of cervical disease associated to HPV in human immunodeficiency infected women under highly active antiretroviral therapy. Infect Agent Cancer. 2009;4:9.
26. Feng R, Zhang H, Qiao YL. HPV prevalence and cervical intraepithelial neoplasia among HIV-infected women in Yunnan Province, China. Unpublished. 2012.
27. Sahasrabuddhe VV, Bhosale RA, Joshi SN, et al. Prevalence and predictors of colposcopic-histopathologically confirmed cervical intraepithelial neoplasia in HIV-infected women in India. PLoS ONE [Electronic Resource]. 2010;5(1):e8634.
28. de Andrade AC, Luz PM, Velasque L, et al. Factors associated with colposcopy-histopathology confirmed cervical intraepithelial neoplasia among HIV-infected women from Rio De Janeiro, Brazil. PLoS ONE [Electronic Resource]. 2011;6(3):e18297.
29. Patrelli TS, Gizzo S, Peri F, et al. Impact of Highly Active Antiretroviral Therapy on the Natural History of Cervical Precancerous Lesions: A 17-Year Institutional Longitudinal Cohort Study. Reprod Sci. 2013;21(7):837-45.
30. Kitchener H, Nelson L, Adams J, et al. Colposcopy is not necessary to assess the risk to the cervix in HIV-positive women: an international cohort study of cervical pathology in HIV-1 positive women. Int J Cancer. 2007;121(11):2484-91.
31. Adler DH, Kakinami L, Modisenyane T, et al. Increased regression and decreased incidence of human papillomavirus-related cervical lesions among HIV-infected women on HAART. AIDS. 2012;26(13):1645-52.
32. Firnhaber C, Westreich D, Schulze D, et al. Highly active antiretroviral therapy and cervical dysplasia in HIV-positive women in South Africa. Journal of the International AIDS Society. 2012;15(2):17382.
33. Kreitchmann R, Bajotto H, da Silva DA, Fuchs SC. Squamous intraepithelial lesions in HIV-infected women: prevalence, incidence, progression and regression. Arch Gynecol Obstet. 2013;288(5):1107-13.
34. Sirera G, Videla S, Lopez-Blazquez R, et al. Highly active antiretroviral therapy and incidence of cervical squamous intraepithelial lesions among HIV-infected women with normal cytology and CD4 counts above 350 cells/mm3. Journal of Antimicrobial Chemotherapy. 2008;61(1):191-4.

35. Soncini E, Zoncada A, Condemi V, Antoni AD, Bocchialini E, Soregotti P. Reduction of the risk of cervical intraepithelial neoplasia in HIV-infected women treated with highly active antiretroviral therapy. Acta Bio-Medica de l Ateneo Parmense. 2007;78(1):36-40.
36. Lehtovirta P, Finne P, Nieminen P, et al. Prevalence and risk factors of squamous intraepithelial lesions of the cervix among HIV-infected women - a long-term follow-up study in a low-prevalence population. Int J STD AIDS. 2006;17(12):831-4.
37. Heard I, Potard V, Costagliola D. Limited impact of immunosuppression and HAART on the incidence of cervical squamous intraepithelial lesions in HIV-positive women. Antiviral Therapy. 2006;11(8):1091-6.
38. Schuman P, Ohmit SE, Klein RS, et al. Longitudinal study of cervical squamous intraepithelial lesions in human immunodeficiency virus (HIV)-seropositive and at-risk HIV-seronegative women. Journal of Infectious Diseases. 2003;188(1):128-36.
39. Ellerbrock TV, Chiasson MA, Bush TJ, et al. Incidence of cervical squamous intraepithelial lesions in HIV-infected women. Jama. 2000;283(8):1031-7.
40. Clifford GM, Franceschi S, Keiser O, et al. Immunodeficiency and the risk of cervical intraepithelial neoplasia 2/3 and cervical cancer: A nested case-control study in the Swiss HIV cohort study. Int J Cancer. 2016;138(7):1732-40.
41. Zeier MD, Botha MH, van der Merwe FH, et al. Progression and persistence of low-grade cervical squamous intraepithelial lesions in women living with human immunodeficiency virus. J Low Genit Tract Dis. 2012;16(3):243-50.
42. Omar T, Schwartz S, Hanrahan C, et al. Progression and regression of premalignant cervical lesions in HIV-infected women from Soweto: a prospective cohort. AIDS. 2011;25(1):87-94.
43. Kim SC, Messing S, Shah K, Luque AE. Effect of highly active antiretroviral therapy (HAART) and menopause on risk of progression of cervical dysplasia in human immune-deficiency virus- (HIV-) infected women. Infectious Diseases in Obstetrics & Gynecology. 2013;2013:784718.
44. Paramsothy P, Jamieson DJ, Heilig CM, et al. The effect of highly active antiretroviral therapy on human papillomavirus clearance and cervical cytology. Obstetrics & Gynecology. 2009;113(1):26-31.
45. Minkoff H, Ahdieh L, Massad LS, et al. The effect of highly active antiretroviral therapy on cervical cytologic changes associated with oncogenic HPV among HIV-infected women. AIDS. 2001;15(16):2157-64.
46. Lillo FB, Ferrari D, Veglia F, et al. Human papillomavirus infection and associated cervical disease in human immunodeficiency virus-infected women: effect of highly active antiretroviral therapy. Journal of Infectious Diseases. 2001;184(5):547-51.
47. Massad LS, Evans CT, Minkoff H, et al. Natural history of grade 1 cervical intraepithelial neoplasia in women with human immunodeficiency virus. Obstet Gynecol. 2004;104(5 Pt 1):1077-85.
48. Heard I, Tassie JM, Kazatchkine MD, Orth G. Highly active antiretroviral therapy enhances regression of cervical intraepithelial neoplasia in HIV-seropositive women. AIDS. 2002;16(13):1799-802.
49. Del Mistro A, Bertorelle R, Franzetti M, et al. Antiretroviral therapy and the clinical evolution of human papillomavirus-associated genital lesions in HIV-positive women. Clinical Infectious Diseases. 2004;38(5):737-42.
50. Chen YC, Li CY, Liu HY, Lee NY, Ko WC, Ko NY. Effect of antiretroviral therapy on the incidence of cervical neoplasia among HIV-infected women: a population-based cohort study in Taiwan. Aids. 2014;28(5):709-15.
51. Guiguet M, Boue F, Cadranel J, Lang JM, Rosenthal E, Costagliola D. Effect of immunodeficiency, HIV viral load, and antiretroviral therapy on the risk of individual malignancies (FHDH-ANRS CO4): a prospective cohort study. Lancet Oncol. 2009;10(12):1152-9.

52. Ki EY, Kim HE, Choi Y-J, Park J-S, Kang CS, Lee A. Comparison of the Cobas 4800 HPV Test and the Seeplex HPV4A ACE with the Hybrid Capture 2 Test. International Journal of Medical Sciences. 2013;10(2):119-23.
53. Comar M, Iannacone MR, Casalicchio G, McKay-Chopin S, Tommasino M, Gheit T. Comparison of Hybrid Capture II, Linear Array, and a Bead-Based Multiplex Genotyping Assay for Detection of Human Papillomavirus in Women with Negative Pap Test Results and Atypical Squamous Cells of Undetermined Significance. Journal of Clinical Microbiology. 2012;50(12):4041-6.
54. Liu SS, Leung RC, Chan KK, Cheung AN, Ngan HY. Evaluation of a newly developed GenoArray human papillomavirus (HPV) genotyping assay and comparison with the Roche Linear Array HPV genotyping assay. J Clin Microbiol. 2010;48(3):758-64.
55. Chakravarty J, Chourasia A, Thakur M, Singh AK, Sundar S, Agrawal NR. Prevalence of human papillomavirus infection & cervical abnormalities in HIV-positive women in eastern India. Indian J Med Res. 2016;143(1):79-86.
56. Katumba AC, Reji E, Gitau T, C. F. World Health Organisation staging, adherence to HAART and abnormal cervical smears amongst HIV-infected women attending a government hospital in Johannesburg, South Africa. Southern African Journal of Infectious Diseases. 2016;31(4):112-8.
57. Liu E, McCree R, Mtisi E, et al. Prevalence and risk factors of cervical squamous intraepithelial lesions among HIV-infected women in Dar es Salaam, Tanzania. Int J STD AIDS. 2016;27(3):219-25.
58. Prabha Devi K, Bindhu Priya N. Conventional Pap Smear Screening in HIV Seropositive Women in South India. Journal of Obstetrics and Gynaecology of India. 2013;63(1):55-8.
59. Marchetti G, Comi L, Bini T, et al. HPV Infection in a Cohort of HIV-Positive Men and Women: Prevalence of Oncogenic Genotypes and Predictors of Mucosal Damage at Genital and Oral Sites. J Sex Transm Dis. 2013;2013:915169.
60. Chalermchockcharoenkit A, Chayachinda C, Thamkhantho M, Komoltri C. Prevalence and cumulative incidence of abnormal cervical cytology among HIV-infected Thai women: A 5.5-year retrospective cohort study. BMC Infectious Diseases. 2011;11(8).
61. Drogoul-Vey MP, Marimoutou C, Robaglia-Schlupp A, et al. Determinants and evolution of squamous intraepithelial lesions in HIV-infected women, 1991-2004. AIDS Care - Psychological and Socio-Medical Aspects of AIDS/HIV. 2007;19(8):1052-7.
62. Mangclaviraj S, Kerr SJ, Chaithongwongwatthana S, et al. Nadir CD4 count and monthly income predict cervical squamous cell abnormalities in HIV-positive women in a resource-limited setting. Int J STD AIDS. 2008;19(8):529-32.
63. Jaquet A, Horo A, Ekouevi DK, et al. Risk factors for Cervical Intraepithelial Neoplasia in HIV-infected women on antiretroviral treatment in Cote d'Ivoire, West Africa. PLoS ONE. 2014;9(3).
64. Oliveira PM, Oliveira RPC, Travessa IEM, Gomes MVC, dos Santos MLJ, Grassi MFR. Prevalence and risk factors for cervical intraepithelial neoplasia in HIV-infected women in Salvador, Bahia, Brazil. Sao Paulo Medical Journal. 2010;128(4):197-201.
65. Gedefaw A, Astatkie A, Tessema GA. The prevalence of precancerous cervical cancer lesion among HIV-infected women in southern Ethiopia: a cross-sectional study. PLoS One. 2013;8(12):e84519.
66. Curry CL, Sage YH, Vragovic O, Stier EA. Minimally abnormal pap testing and cervical histology in hiv-infected women. Journal of Women's Health. 2012;21(1):87-91.
